# An effective uranium removal using diversified synthesized cross-linked chitosan bis-aldehyde Schiff base derivatives from aqueous solutions

**DOI:** 10.1007/s11356-022-23856-2

**Published:** 2022-11-05

**Authors:** Amira Hamed, Ahmed Orabi, Hend Salem, Doaa Ismaiel, Gamal Saad, Ismail Abdelhamid, Ahmed Elwahy, Maher Elsabee

**Affiliations:** 1 Chemistry Department, Faculty of Science, Cairo University, Cairo, 12613 Egypt; 2 Nuclear Materials Authority, El-Maadi, P.O. Box 530, Cairo, Egypt

**Keywords:** Cross-linked chitosan, Adsorption, Uranium, Removal, Waste solutions

## Abstract

Three new cross-linked chitosan derivatives were yielded through intensification of chitosan with diverse types of *bis*-aldehydes. The prepared cross-linked chitosan was characterized by FTIR, ^1^H NMR, XRD, and TGA techniques. TGA indicated an improvement in thermal stability of the cross-linked chitosan compared with pure chitosan. Batch adsorption experiments showed that the three novel cross-linked chitosan *bis*-aldehyde derivatives possessed good adsorption capacity against U(VI) in the order of BFPA > BFB > BODB (adsorption capacity of the three adsorbents for U(VI) reaches 142, 124, and 114 mg/g respectively) and the adsorption isotherm and kinetic were well described by the Langmuir and the pseudo-second-order kinetic model, respectively. In addition, the prepared cross-linked chitosan *bis*-aldehyde derivatives were examined as U(VI) catcher from waste solutions.

**Graphical Abstract:**

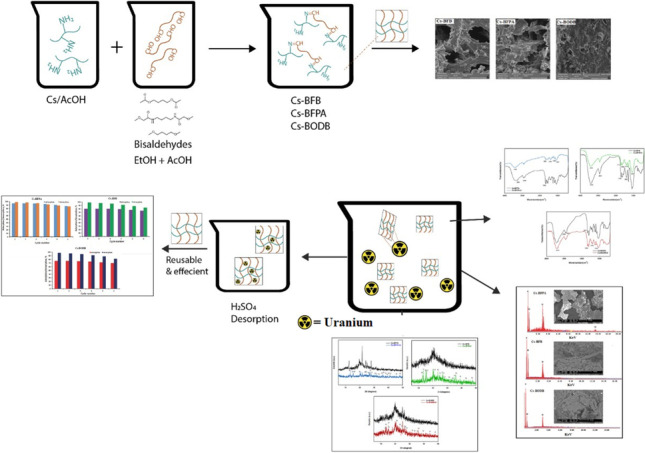

## Introduction

Nuclear power relies heavily on uranium, which is a vital resource (Wang et al. 2014). When it comes to human health and the environment, it is considered a radioactive element that poses a risk (Banerjee et al. 2022). It’s a double-edged sword for nuclear energy and environmental preservation because uranium has both positive and negative effects. That’s why methods to separate and recover uranium from wastewater and polluted ground and ocean are so critical. U(VI) is radioactive, carcinogenic, and toxic, and wastewater containing low U(VI) content will cause irreversible damage to the environment and ecosystem (Banerjee et al. 2022). So, it is of great significance to find an economical and efficient method to separate U(VI) from an aqueous solution.

A safe method for extracting toxic uranium from waste materials in an efficient way is of very great significance. Solvent extraction, adsorption, cationic exchanger, or anion exchanger had been studied for recovery and separation of radionuclides from aqueous wastes (Aydin and Soylak 2007; Ghasemi et al. 2011; Aly and Hamza 2013; Zhu et al. 2018; Orabi et al. 2021). Adsorption technique has been widely used in the separation and preconcentration of elements from environmental samples due to its advantages as less use of organic solvent and less waste build-up, in addition to the ability of ion exchangers and chelating resins to extract U(VI) from a dilute solution (Satilmis et al. 2019). The well-known and widely used type of adsorbents used for the adsorption of uranium is chemisorbents (biopolymer, metal oxides, clay minerals, nanomaterials) (Guerra et al. 2010; Orabi et al. 2016, 2018, 2021; Xue et al. 2017; Yuan et al. 2017; Huang et al. 2018; Zeng et al. 2019; Orabi 2019; Cai et al. 2019; Dacrory et al. 2020). However, the reusability and regeneration of adsorbents have not attained enough attention, or even not been considered (Wang et al. 2017; Liu et al. 2017; Li et al. 2017; Sun et al. 2015). The reusability and regeneration of adsorbents are important factors for the practical application considering the low economic cost and environmental renewability. In addition, natural mineral adsorbents have the maximum adsorption capacity and removal rate for uranium(VI) in the solution with a pH ≥ 5. However, in the solution with strong acidity (pH ˂ 5), the stability of these adsorbents is poor, and it is difficult to maintain the original structure, resulting in poor adsorption performance, and additional surface modification is needed to enhance the removal efficiency of uranium(VI) in highly acidic solution (pH ˂ 5). At present, uranium containing waste solutions mainly comes from uranium mining, and uranium containing wastewater from uranium tailings is often highly acidic (pH ˂ 5) (Pereira et al. 2018). The biopolymers (chitosan, cellulose, and alginate) in particular have gained interest due to their abundance in nature, non-toxicity, and cost-effectiveness in comparison to other materials. Unfortunately, low adsorption capacity limits their application as adsorbents. In order to overcome the disadvantage, researchers have focused on modifying biopolymers in order to achieve high adsorption capacity and improved selectivity. Chitosan (Cs) is an incredible natural polymer and it has evolved as a one-of-a-kind polymer that may be used in many different ways, including as a food additive (Elsabee and Entsar 2013). Cs is a suitable option for a variety of applications because it has a plentiful supply of reactive amino and hydroxyl groups and can undergo a wide range of modifications. These characteristics have enabled Cs to successfully contribute to ecologically sustainable solutions. Cs undergoes reactions with epoxides, anhydrides, or bis-aldehydes during the process of cross-linking, which is a common method for altering Cs (Mi et al. 2001; Hastuti et al. 2016; Zou et al. 2017; Wahba 2020). The Cs’s absorption qualities are improved as a result of cross-linking, which also boosts the functionality that is required, such as the Cs’s tolerance to acid. It was common knowledge that bis-aldehydes might be exploited as precursors for a wide variety of bis (functionalized) organic compounds. These compounds are known to possess pharmacological and therapeutic effects (Sanad et al. 2016). Cross-linked network gel structure was capable of absorbing and retaining large volumes of water without dissolving or destroying their three-dimensional form. Out of all the adsorbents, the organic compounds which have nitrogen, phosphorus, oxygen, and sulfur in their structure are the most effective. One of such efficient organic compounds which is an excellent adsorbent is Schiff base. Schiff base forms a stable complex with heavy metal ions due to the electrostatic interaction between the unshared electron pairs and the nearby electrons of the metal, thus making promising adsorbents (Baran et al. 2018). In connection with the above-mentioned findings coupled with our ongoing interest in searching for promising chitosan-based materials, the present work aims at the preparation of chitosan formulation using the Schiff base reaction between chitosan and three bis-aldehydes containing different functional groups. These new adsorbents exhibited highly selective enhancement towards uranyl ions in the presence of competing ions. The fibrous nature of Cs was intact even after modifications exhibiting favorable application prospects. Limited knowledge is available for the synthesis of Schiff base on Cs surface via three bis-aldehydes containing different functional groups for detection and annihilation of uranyl ions from aqueous solution. The derivatives were characterized by ^1^H NMR, FTIR, ESEM, XRD, and TGA techniques. The specific target was to study their capacity for adsorption and removal of uranium from waste solutions. The analysis and optimization of a variety of process variables that influence the removal process were conducted. It was necessary to conduct kinetics, isotherm, and thermodynamic investigations in order to have a better knowledge of the adsorption process. Also investigated was the possibility of adsorbent regeneration to reduce the cost of the treatment procedure, and, finally, waste materials were subjected to laboratory testing.

## Experimental

### Materials

Chitosan was prepared as described previously (Abdou et al. 2008). Other reagents came from Merck (Germany). *Bis*-aldehydes “*N,N'*-(butane-1,4-diyl)bis(2-(4-formylphenoxy)acetamide), BFPA, butane-1,4-diyl bis(4-formylbenzoate), BFB, and 4,4′-(butane-1, 4-diylbis(oxy))dibenzaldehyde, BODB” were prepared as outlined previously (Elwahy 1999; Mohamed et al. 2018; Abdella et al. 2020).

### Synthesis of Cs-bis-aldehyde Schiff bases (CBASB)

**Fig. 1:**
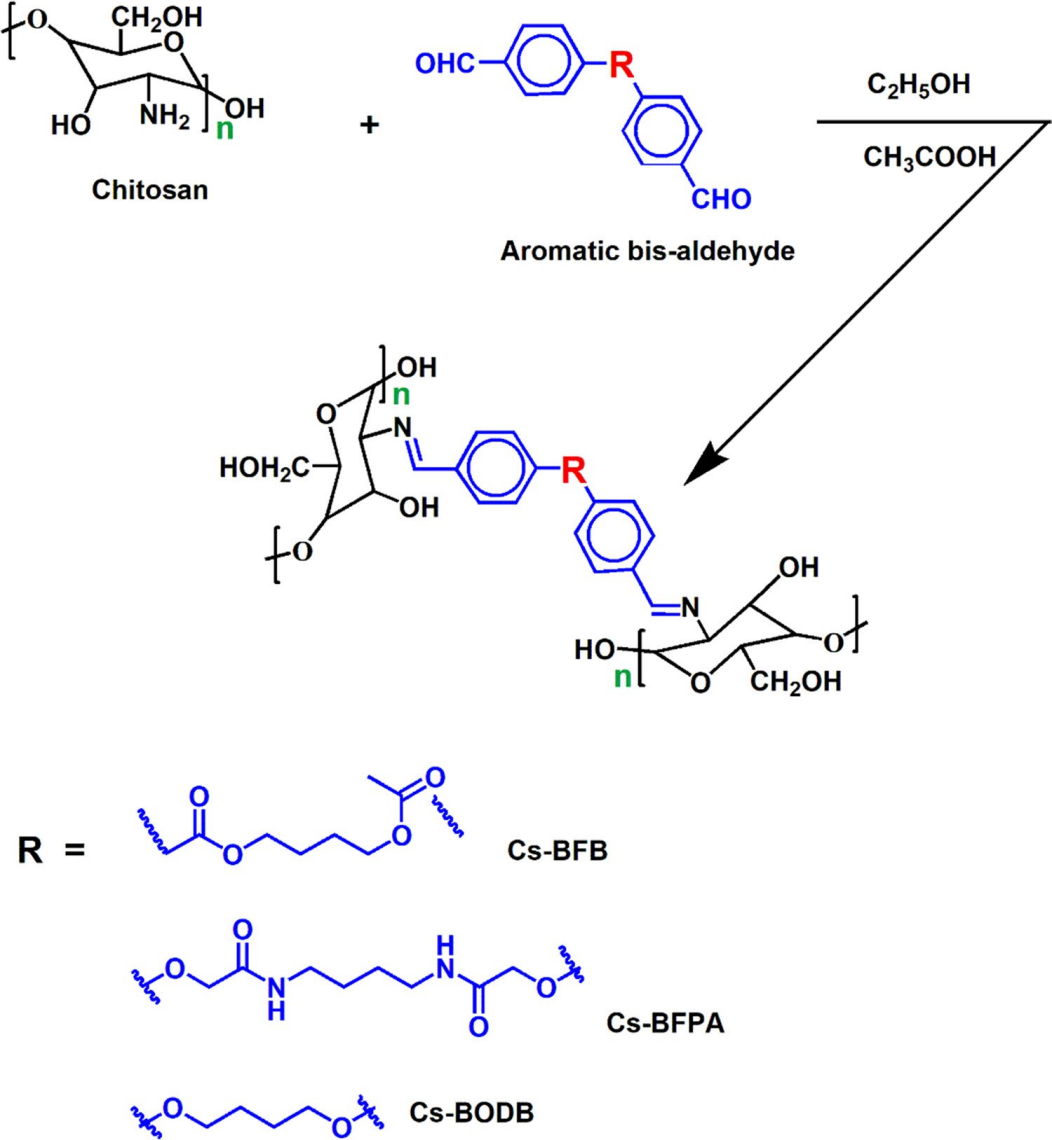
Synthesis of Cs aromatic bis-aldehyde Schiff bases

The formation of Schiff bases of chitosan was carried out in accordance with the steps specified in Fig. 1. At room temperature, 1.0 g of chitosan was dissolved in approximately 100 mL of acetic acid at a concentration of 2.0 percent, followed by the addition of 50 mL of ethanol. The solution of chitosan was slowly given the preset quantities of aromatic bis-aldehyde (0.35 g) that had been dissolved in 10 mL of an ethanol/acetic acid mixture that was 50/50, volume/volume. After 12 h of constant stirring at a temperature of 80 °C, the reaction mixtures were allowed to proceed with their reactions. After the reaction had run its course, the product was isolated by precipitating it with an excess of ethanol and a few drops of NH_4_OH to bring the pH up to 10.0. The precipitate was refined and washed numerous times with anhydrous ethanol to remove the unreacted aldehyde and let it dry at room temperature. In this case, the Schiff bases produced from the reaction of chitosan with BFPA, BFB, and BODB derivatives are coded here as Cs-BFPA, Cs-BFB, and Cs-BODB, respectively.

### Characterization techniques

The morphological features of the chitosan Schiff bases were observed by the scanning electron microscope (Hitachi S-4800, Japan). An FTIR spectrum spectrometer (Nicolet Avatar 360, USA) was utilized to characterize the identifications of material functional groups between 500 and 4000 cm^−1^.

An X-ray powder diffractometer (a Philips MPD Pro) was used to produce X-ray diffractograms of the generated powder samples. 2°/min scanning rate and a scanning diffraction angle of 5° to 80° at 25 °C were used. While running under a 30 mL/min nitrogen flow via a Thermal Analyzer Q 2000, USA, TGA was done between ambient and 600 °C at a dynamic heating rate of 10 °C min^−1^. The particle size, size distribution, and zeta-potential measurements of the Cs and Cs derivatives were determined with Malvern Zetasizer Nano instruments Ltd. (UK) at 25 °C. The measurements were carried out by suspending the investigated materials in deionized water.

### Adsorption performance studies

U(VI) adsorption experiments in an aqueous solution were studied using synthesized Cs-bis-aldehydes (Cs-BFPA, Cs-BFB, and Cs-BODB). The adsorption experiments were carried out with 0.05 g (m) adsorbent in 25 mL (V) U(VI) aqueous solution. The concentration of U(VI) in the solution was determined fluorometrically by using the Uranium Analyzer (Scintrex, Canada). The effects of different factors on adsorption were observed by changing the varying initial adsorbent dose, pH value, time, and U(VI) concentration. The amounts of adsorbed ions *q*_e_ (mg/g), separation factor (SF), and the percentage adsorption on the synthesized Cs-*bis*-aldehyde Schiff bases (CBASB) adsorbents (Ads%) were defined by the next empirical formulas:

1 $$q\mathrm{e}=\left(\frac{{C}_{0}- {C}_{e}}{m}\right)\times V$$

2 $$\mathrm{Ads\%}=\left(\frac{{(C}_{0}- {C}_{e })}{{\mathrm{C}}_{0}}\right)\times 100$$

3 $$\mathrm{SF}=\frac{{K}_{d (U)}}{{K}_{d (M)}}= \frac{qe\left(U\right)\times Ce (M)}{Ce(U)\times qe(M)}$$

where *C*_0_ and *C*_e_ are the concentrations of U(VI) in solution at initial and equilibrium (mg/L). The standard uncertainty of the data was below 5%. Kd is the ratio of the distribution constant (Kd = *q*_e_/*C*_e_). Kd (U) is the distribution constant of uranium and Kd (M) is the distribution constant of interfering metal ions.

### The working liquid wastes

Experiment liquid waste samples (1 L) were sourced from Egypt’s Nuclear Material Authority until they had been modified to work at the desired pH. As you can see in Table 1, we performed chemical analyses (using ICP-OES, Teledyne Technologies) on a variety of samples of liquid waste.

**Table 1:** The chemical constitutions (mg/L) of the waste sample solutions

Sample	Ca^2+^	Mg^2+^	Mn^2+^	Pb^2+^	Cu^2+^	Fe^3+^	Al^3+^	Zn^2+^	Cd^2+^	U^6+^	Cl^−^
a	144.0	45.0	13.4	8.6	12.0	600.0	160.0	13.0	8.9	76.0	768.0
b	170.6	29.7	15.3	11.4	10.5	135.0	182.8	11.4	0.3	54.0	97.0

## Results and discussion

### Characterization

#### Fourier transform infrared spectroscopy (FTIR)

**Fig. 2:**
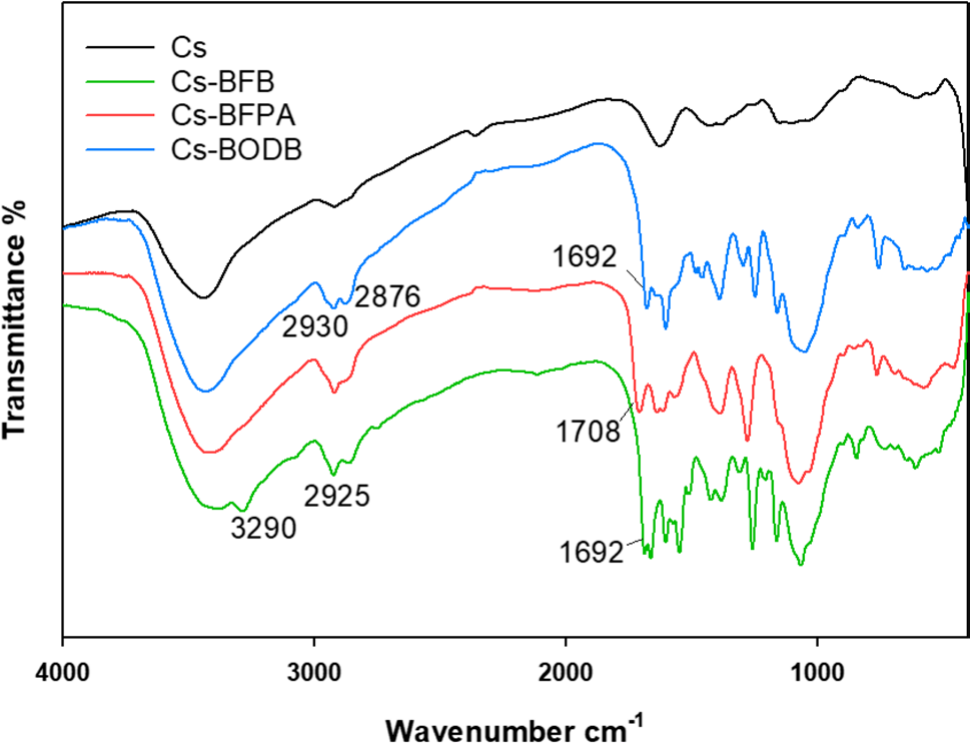
FTIR spectra of chitosan (Cs), Cs-BFB, Cs-BFPA, and Cs-BODB

The FTIR spectrum (Fig. 2) compares Cs-BFPA, Cs-BFB, and Cs-BODB SBDs to chitosan (Cs). Cs’s NH, OH, and NH_2_ groups stretching vibrations were shown at 3000–3500 cm^−1^. Additionally, the well-known Cs’s C = O, C-H bending, the glucopyranoside ring stretching, and the C-N deformation of amino groups were substantiated by the bands at 1654, 2931, 1160, and 1426 cm^−1^, respectively (Salama et al. 2015).

Bands from 1647 to 1688 cm^−1^ in the FTIR spectrum of chitosan were attributed to the imine C = N group, supporting the interaction between chitosan and aromatic bis-aldehydes. “Aromatic C-H in-plane bending, and C = C” in Schiff base derivatives (SBDs) were substantiated via the bands at roughly 1056 and 1500 cm^−1^, respectively (Timur and Paşa 2018). While asymmetric and symmetric C-H stretching are attributed to the bands at 2895 and 2927 cm^−1^, respectively. (Salama et al. 2015). The ester and amide C = O moieties stretching vibrations in SBDs are shown at 1706 and 1719 cm^−1^ prominent peaks (Abdella et al. 2020).

#### ***Proton nuclear magnetic resonance (***.^***1***^***H NMR)***

**Fig. 3:**
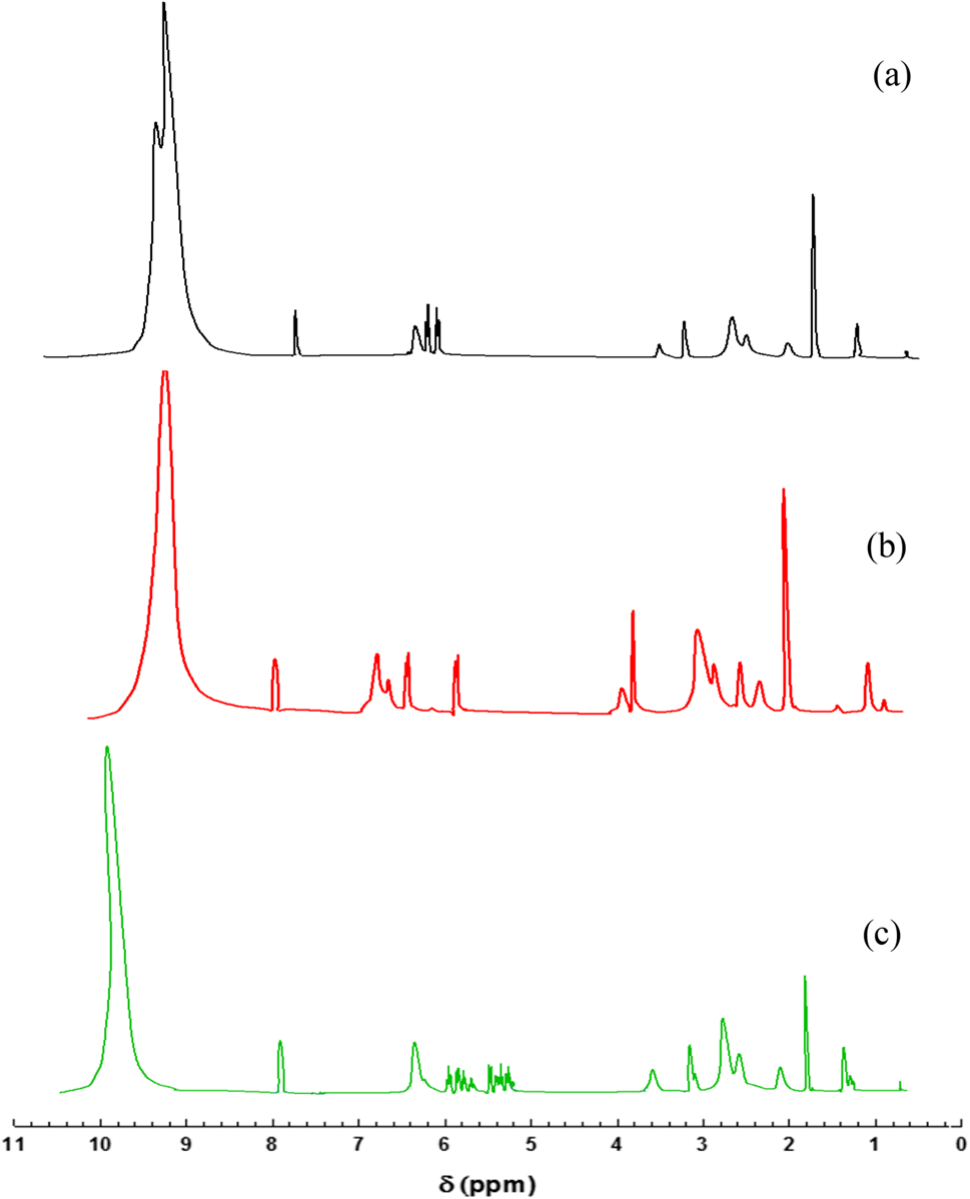
^1^H NMR spectra of (**a**) Cs-BFB, (**b**) Cs-BFPA, and (**c**) Cs-BODB

^1^H NMR spectra (Fig. 3) demonstrate SBDs derivatives structure. For instance, the ^1^H NMR pattern of Cs was observed in all the spectra as follows: N-acetyl protons of the pyranose ring, multiplet signals of H3, H4, H5, H6, H6′, and two singlets corresponding to H2 protons were justified at 1.9, 3.3–4.4, and 3.0 ppm, respectively.

Additionally, the reaction of Cs with the aromatic bis-aldehydes was substantiated via the signals at 7.0–8.3, 10.0 ppm that are typical of an aromatic ring, and imine group protons, respectively. Furthermore, signals of aromatic bis-aldehydes signals can be observed in all three charts. For instance, a 4.13 ppm signal was correlated with 4H of CH_2_–O in Cs-BODB spectrum, 4.37 ppm was ascribed to the -CH_2_ (s, 4H, 2 COOCH_2_) protons adjacent to the ester group in Cs-BFB. The 4H of CH_2_N and OCH_2_ presence in Cs-BFBA spectrum were substantiated at 2.8 and 4.58 ppm, respectively (Elwahy 1999; Mohamed et al. 2018; Abdella et al. 2020).

#### X-ray diffractograms (XRD)

**Fig. 4:**
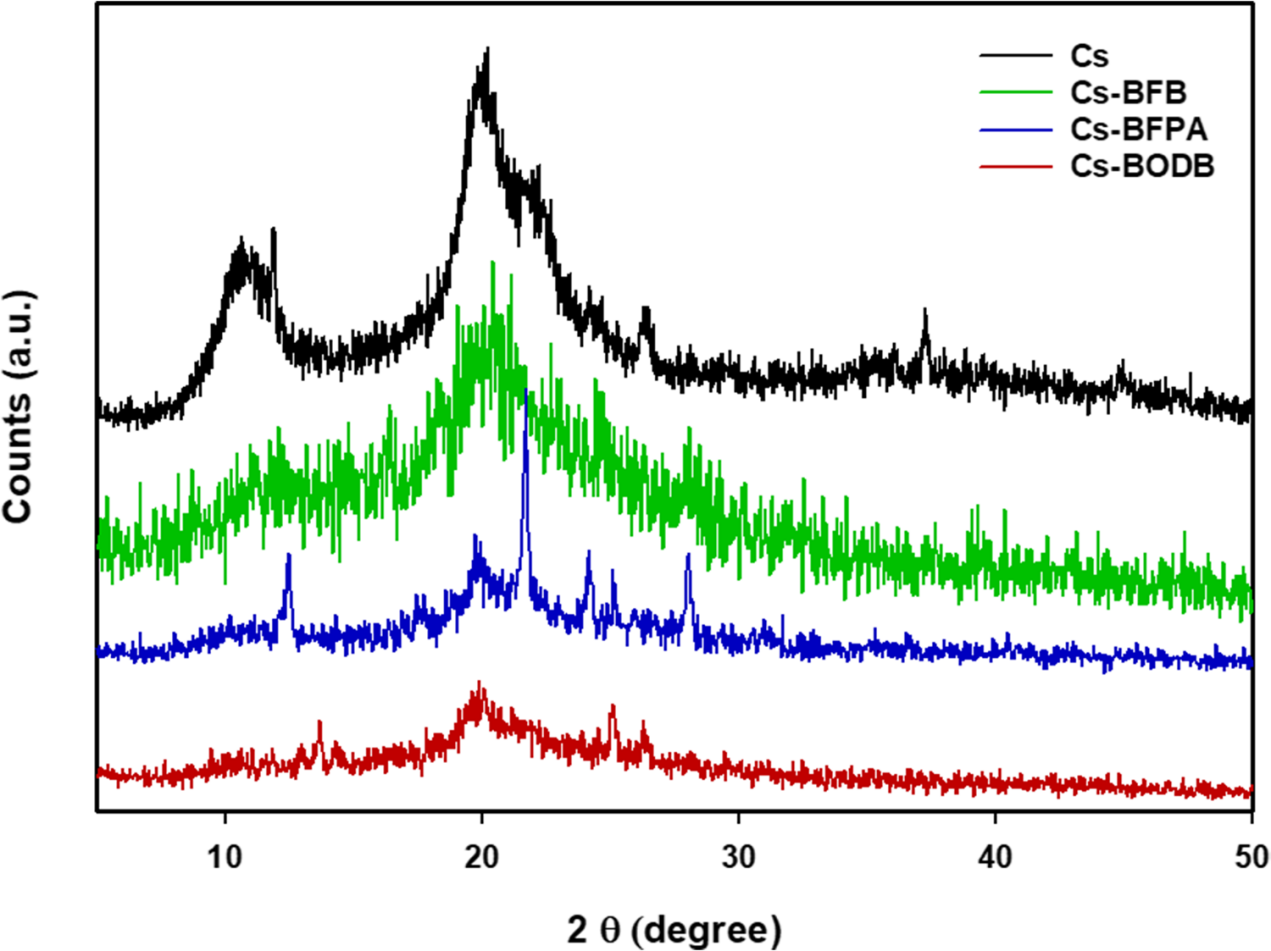
XRD patterns of chitosan and CBASB derivatives

In Fig. 4, CBASB derivatives and chitosan XRD patterns are shown in comparison to one another. Cs displayed two distinct peaks at 20.1° and 10.3° in its pattern. Because the disappearance of the peak at 2θ = 10.3° and the typical peak at 2θ = 20.1° is wider and less intense for Cs Schiff bases than it is for Cs, this shows that Cs Schiff bases have a more amorphous character than Cs. The inclusion of bis-aldehyde cross-linkers in chitosan may have disrupted the ordered structure, resulting in a less crystalline form for Cs Schiff bases.

#### Scanning electron microscope (SEM)

**Fig. 5:**
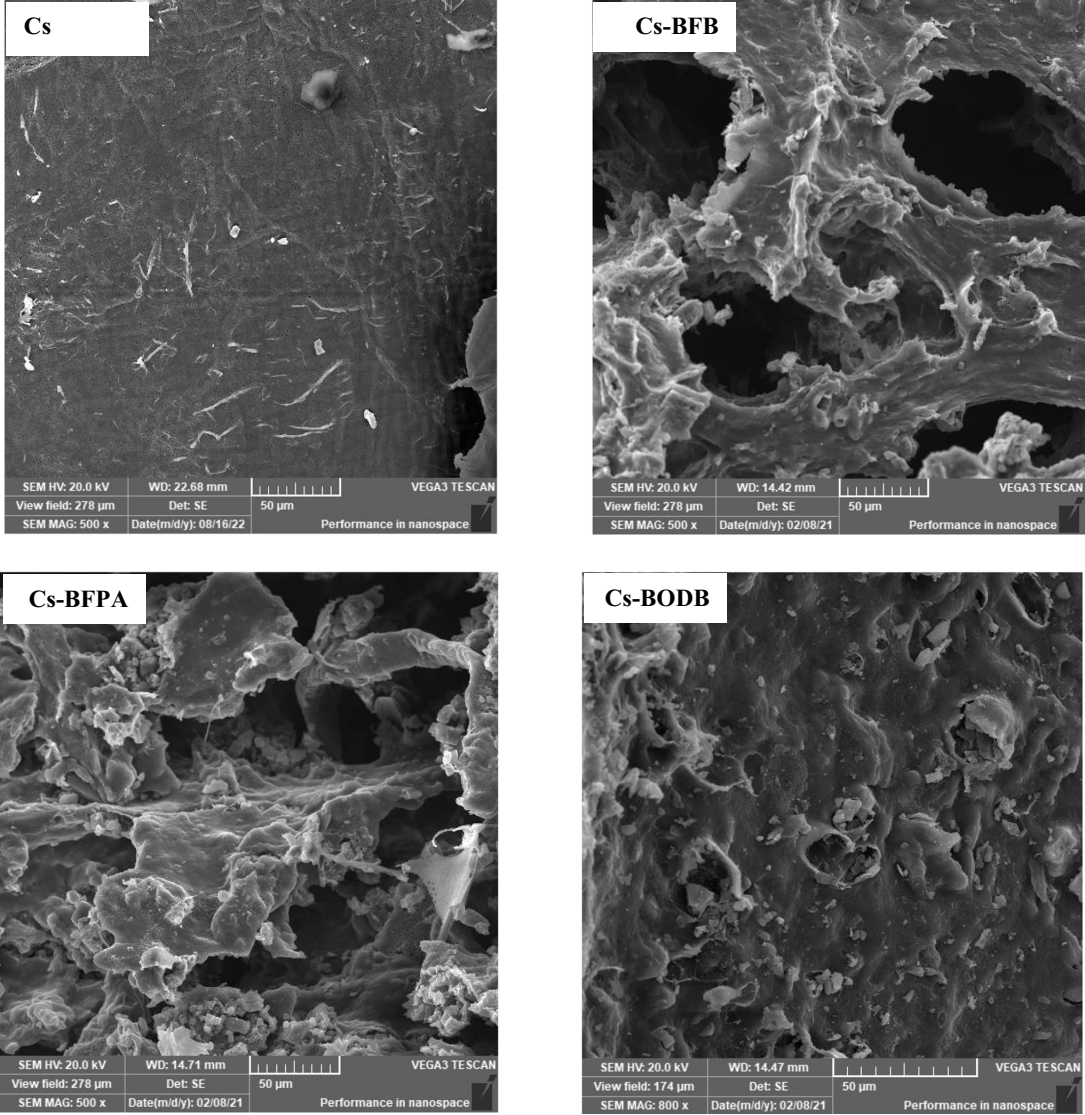
SEM micrograph of the Cs, Cs-BFB, Cs-BFPA, and Cs-BODB derivatives

The scans revealed substantial surface morphological changes between Cs and different *bis*-aldehyde Schiff bases. For instance, Cs revealed several appendages on the smooth-looking surface while the SEM photographs of representative examples of the Cs Schiff bases (Fig. 5) revealed a relatively rough surface with an uneven structure, suggesting that the Cs derivatives exhibited somewhat an amorphous feature attributed to the inclusion of *bis*-aldehyde cross-linkers. By comparing the Cs Schiff base derivatives with different kinds of *bis*-aldehydes, it could be seen that the porous size differs with the different kinds of cross-linker, indicating that the type of bis-aldehyde cross-linking agent in the system plays an extremely important role in determining the pore size. On careful examination (Fig. 5), one can notice that the BFB leads to the formation of a less dense pore structure of partially cross-linked chitosan compared with that of BFPA and BODB. This might be attributed to the rigid structure of the ester group in the BFB cross-linker.

#### Thermogravimetric (TG) and differential thermogravimetric (DTG) analysis

**Fig. 6:**
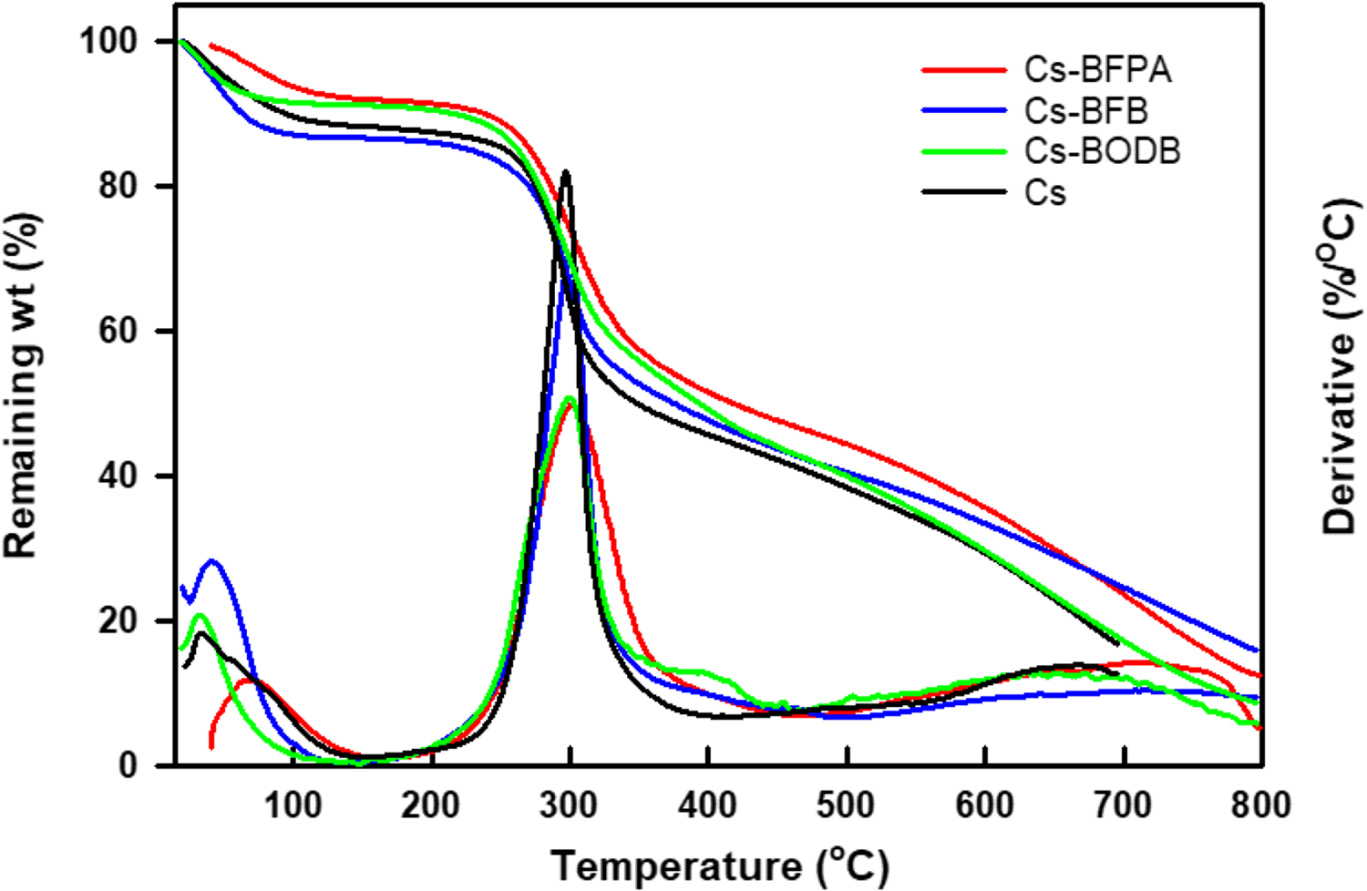
Thermogravimetric curves (TGA and DTGA) of chitosan and CBASB derivatives (Cs-BODB, Cs-BFPA, and Cs-BFB)

Figure 6 shows the TG/DTG curves of chitosan and CBASB derivatives. It is obvious that all the tested samples gave various stages of mass loss. As seen in Fig. 6, native chitosan showed two main stages of mass loss. “The first stage of mass loss began at about 60 °C, almost 11.0% occurs, which was attributed to the release of the loss of residual or physically adsorbed and bound water. The second stage, which started at about 250 °C and reached a maximum of 297 °C with a mass loss of about 51.5%, was described as the chain scission and degradation of the chitosan chain. TG curves of chitosan derivatives also showed two main distinct windows of mass loss. The first one, occurring at lower temperatures compared with chitosan, was assigned to the moisture loss from the Cs network structure, which is obvious because of the Cs hydrophilicity. The second stage of mass loss, occurring at a slightly higher temperature compared with chitosan, was associated with the degradation of chitosan base polymer and the imine cross-linkers moieties attached to the chitosan. Based on the onset degradation temperature (*T*_o_) and the maximum degradation temperature (*T*_max_), it can be seen that the thermal stability of the chitosan derivatives slightly increased relative to that of the pure chitosan slightly increased. The thermal stability increases in the following order: Cs-BFPA > Cs-BFB > Cs-BODB > Cs. At the same time, it can be seen that the mass loss at this second stage is lower for chitosan derivatives compared with pure chitosan. Furthermore, the carbonaceous residue of the chitosan derivatives is greater than that of pure chitosan. These results support the formation of the cross-linked structure of chitosan via reaction with *bis*-aldehydes.

The measured surface properties of chitosan and CBASB derivatives, such as pore diameter, pore volume, and BET surface area, are summarized in Table 2. The results demonstrated that the addition of *bis*-aldehyde Schiff bases improved the surface characteristics, including enhancement of the BET surface area, pore diameter, and pore volume. This is consistent with the observations from SEM.

**Table 2:** Pore structure parameters of chitosan and CBASB derivatives

Sample	BET surface area (m^2^g^−1^)	Pore volume (cm^−1^ g^−1^)	Average pore size (nm)
Cs	**25.4794**	**0.0469**	**1.6643**
Cs-BFPA	**49.3752**	**0.1014**	**1.8823**
Cs-BFB	**46.7285**	**0.0959**	**1.7814**
Cs-BODB	**43.1872**	**0.0793**	**1.7159**

### Adsorption performance study

#### Effect of pH

The pH value of the solution will directly affect the valence or existence state of U(VI) ions as well as the properties of the CBASB’s active sites, which are both crucial in the conservation of U(VI) (Zeng et al. 2017), so the pH value of the solution is an important factor that affects the combined process of uranium with CBASB derivatives’ (Cs-BODB, Cs-BFPA, and Cs-BFB) adsorbents in aqueous solution. Under strongly acidic conditions, the stability of adsorbents decreases, while under high pH value, uranium will undergo a hydrolysis reaction, and its binding ability to the adsorbed materials (Cs-BODB, Cs-BFPA, and Cs-BFB) will decrease. Consequently, the pH value of the solution in this part is controlled between 1.0 and 5.0. In all experiments, the U(VI) concentration, the sample volume, and the adsorbent dose were 100 mg/L, 25 mL, and 0.05 g, respectively. All samples were placed in a shaker for 60 min. at 25 °C.

**Fig. 7:**
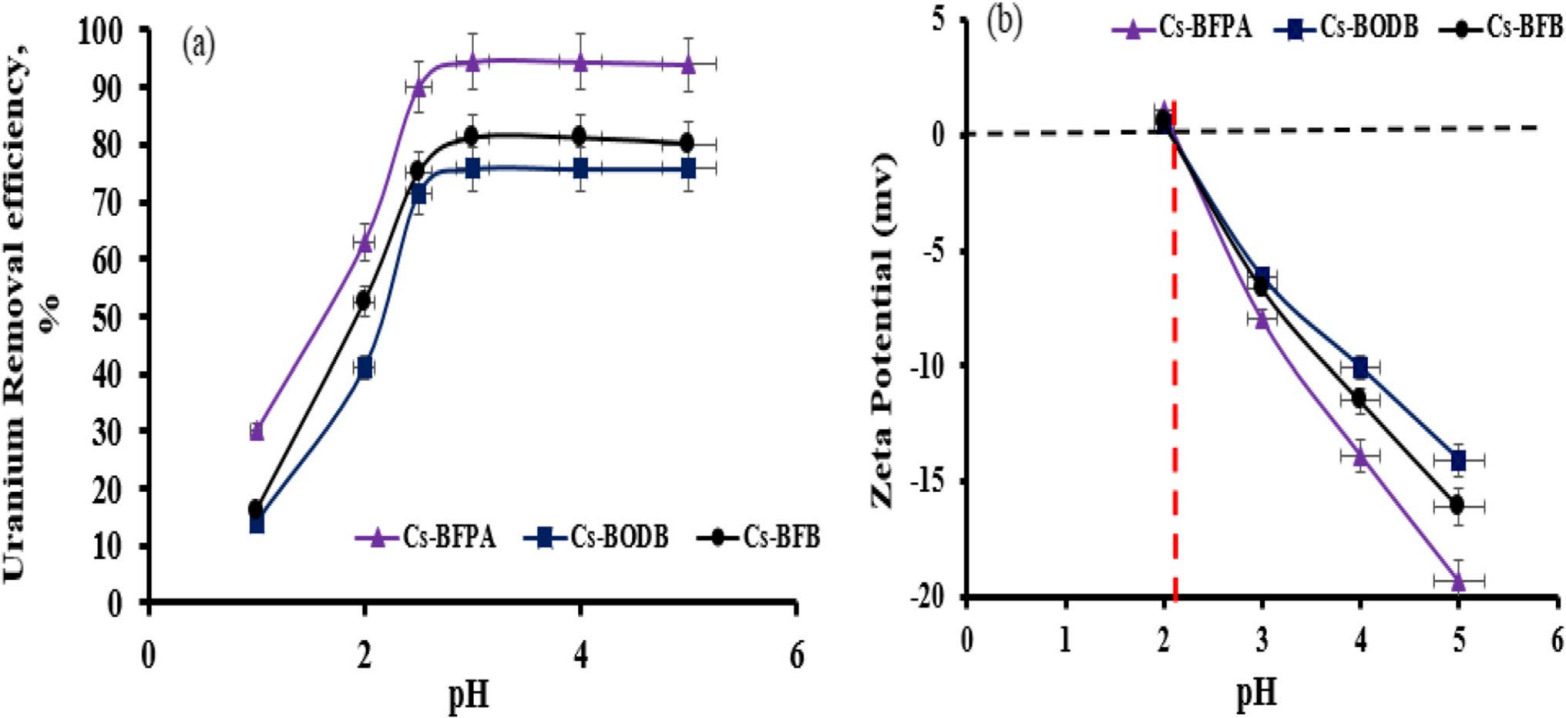
Effect of initial pH on U(VI) adsorption (**a**) and the surface charge (**b**) of CBASB derivatives

As can be seen from Fig. 7a, the pH value in the range of 1.0–5.0, the adsorption % of the three Cs-*bis*-aldehyde Schiff bases derivatives on U(VI) increases with the increase of solution pH value. Because H^+^ ions (from the acidic solution) compete with the Cs-*bis*-aldehydes adsorption sites at low pH levels, hence, U(VI) adsorption reduces (Choppin 2006; Khani et al. 2006; Khawassek et al. 2018; Hussein et al. 2019; Orabi et al. 2021). In addition, increasing acid concentration (very low pH) leads to enhancing the salt effect and consequently, the adsorption process is encountered with some restrictions. Also, the crystals of salt occupy the superficial area of the adsorbents at high salt concentrations, which diminish the adsorbent available to interact with the analytes and play a very negative role by decreasing the recovery. The same behavior agrees with that reported earlier using other adsorbents (Hosseini-Bandegharaei et al. 2013; Fouad et al. 2019; Orabi et al. 2019). The absorption on three adsorbents of U(VI) increased when the pH of the adsorption media raised and reached a maximum at pH 3–5. The increased absorption of U(VI) may be attributed to a decrease in the protonation of the adsorbents’ surfaces at higher pH levels. In addition, free cationic species of uranium (UO_2_^2+^, (UO_2_)(OH)_2_^2+^, UO_2_OH ^+^, and (UO_2_)_3_(OH)_5_^+^) predominate in this region and enhance the metal ions to be extracted (Choppin 2006; Khani et al. 2006; Khawassek et al. 2018; Hussein et al. 2019; Orabi et al. 2021). In order to better study the adsorption performance of CBASB derivatives of U(VI), the pH value of the experimental system was set at 3 in the follow-up study in this paper. At this optimum pH, the adsorption % of uranium is 94.5%, 81.0%, and 75.8% for Cs-BFPA, Cs-BFB, and Cs-BODB, respectively (Fig. 7). Subsequently, the adsorption efficiencies of the new adsorbents for uranium extraction process have been compared to conventional Cs. As expected, the new adsorbents allow a higher separation efficiency than Cs (40% in the same conditions). This is due to more chelating groups (C = O, NH, and C-O) in the new adsorbents than in Cs.

Evaluation of zero point charge (PZC) of Cs-BFPA, Cs-BFB, and Cs-BODB adsorbents is the pH where the surface charge density becomes equivalent to zero. The variation of surface charge of these adsorbents with pH is shown in Fig. 7b. The zero charge points of Cs-BFPA, Cs-BFB, and Cs-BODB adsorbents are 2.15, 2.15, and 2.2, respectively, indicating that, after this pH, the surfaces of the three adsorbents are negatively charged, facilitating the adsorption process of the materials. When the pH value (pH < pHzpv) is low, the adsorbents are protonated, and there is electrostatic repulsion between adsorbents and uranium(VI), which may be an important factor leading to small adsorption capacity. As the pH value (pH > pHzpv) increases, the electrostatic repulsion between the adsorbent and uranium(VI) decreases, so the adsorption capacity increases.

**Fig. 8:**
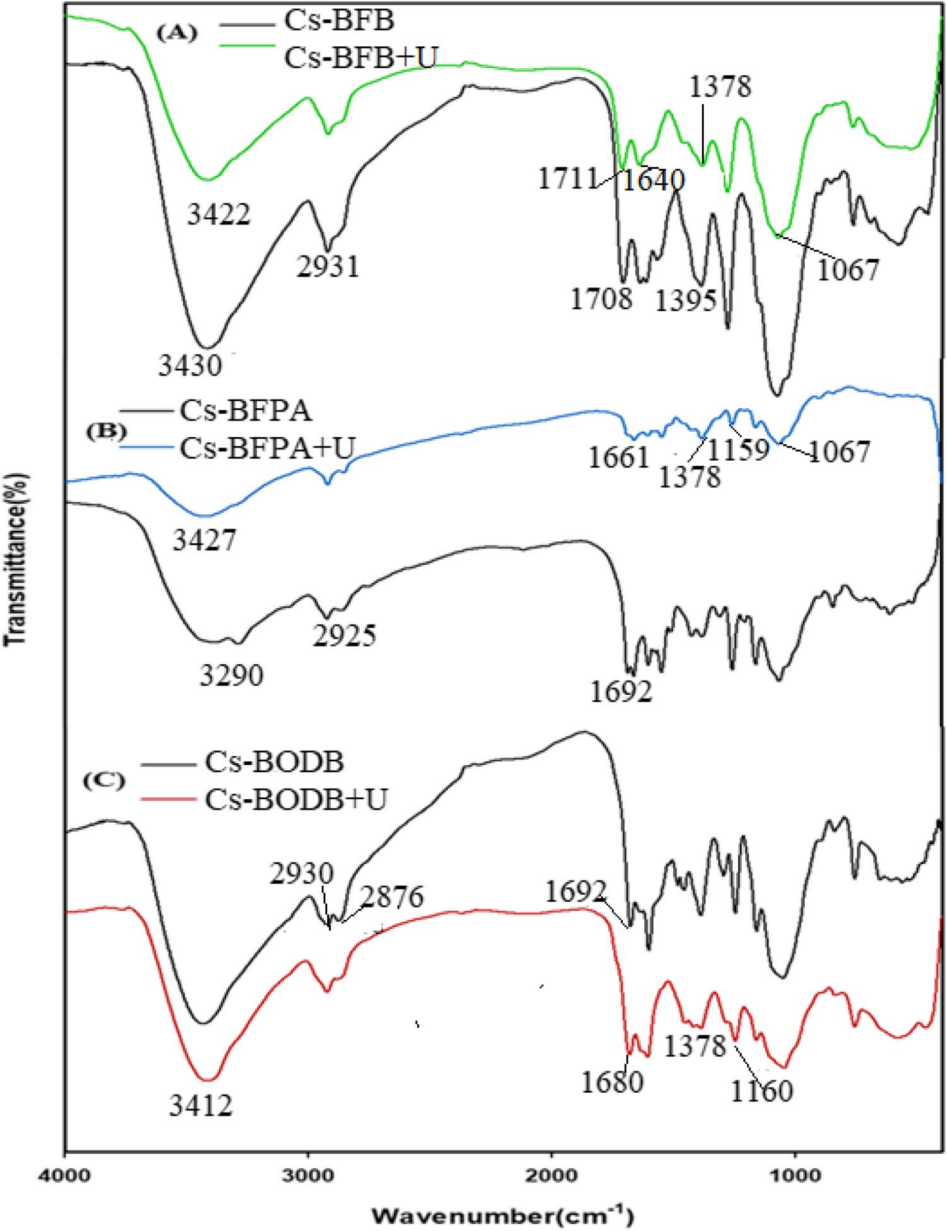
FTIR spectra of U complexation with CBASB derivatives

It can be observed from Fig. 8 that the main difference between CBASB derivatives (Fig. 2) and its complexation with U(VI) (Fig. 8) was the shift of some bands, which were observed due to interaction with metal ions. The bands belonging to C-N and C-O units of Cs-BFPA were shifted and reduced from 1147 to 1338 cm^−1^, and 1056 cm^−1^, to the values 1159–1378 cm^−1^, and 1067 cm^−1^ (Fig. 8B), respectively. In addition, the C = N band was shifted from 1665 to 1661 cm^−1^. Also, the band of C = O has disappeared after its complexation with U(VI). In the case of Cs-BFB adsorbent and its complexation with U(VI) (Fig. 8A), their characteristic bands of C = O and C = N were reduced and shifted from 1719 and 1647 to 1711 and 1640 cm^−1^ (Fig. 8A), respectively. In addition, C-N and C-O bands were shifted from 1338 and 1056 to 1378 and 1067 cm^−1^, respectively. Finally, some frequency shifts were seen in the peaks that corresponded to C = N and C–O ether when Cs-BODB was loaded with uranium. These shifts in frequency may be interpreted as evidence of the sorption of uranium onto the Cs-BODB sorbent that was understudied (Fig. 8C), where they were reduced and shifted from 1688 and 1170 to 1680 and 1160 cm^−1^ (Fig. 8C), respectively.

**Fig. 9:**
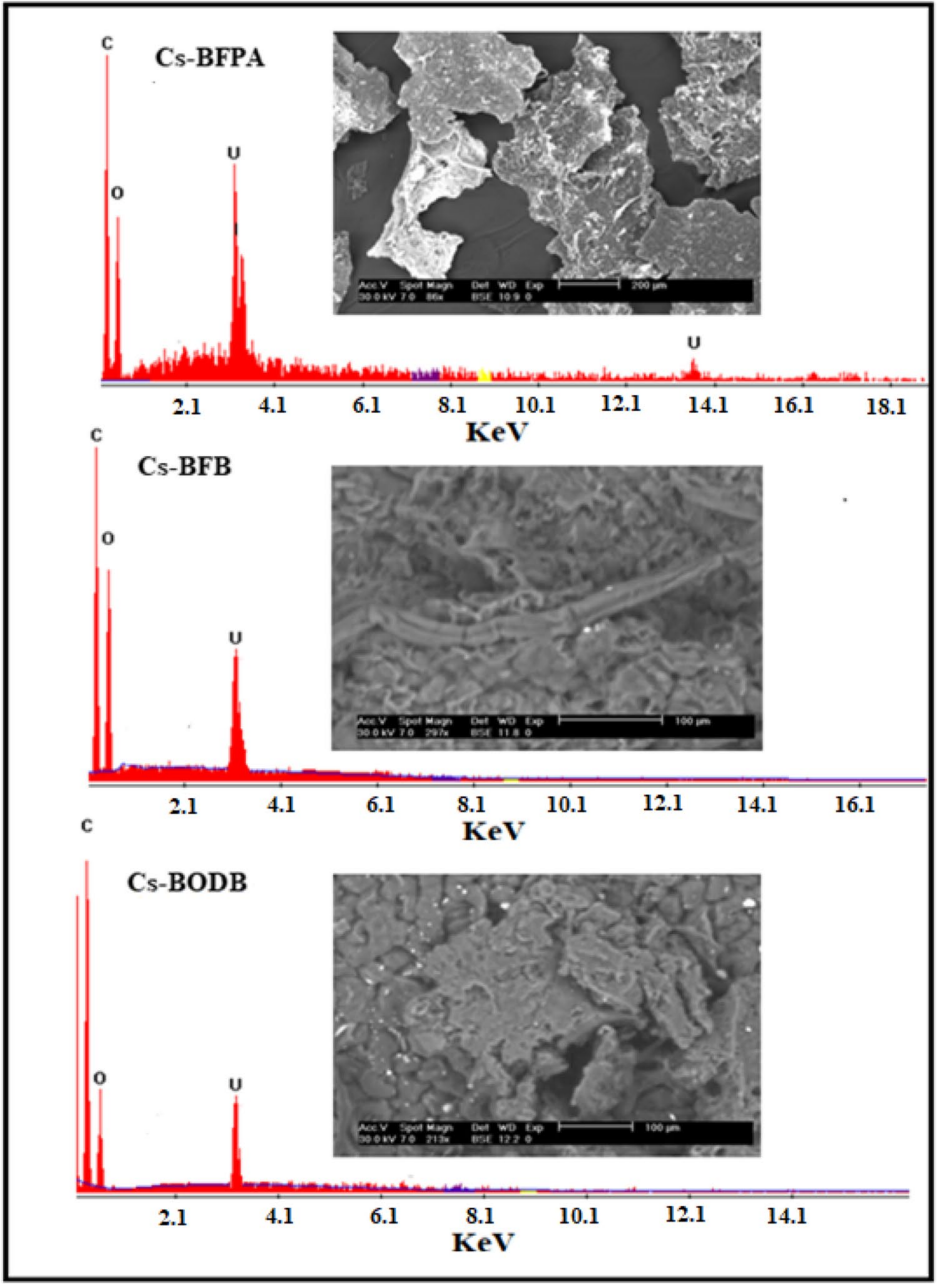
SEM and EDX spectra of U complexation with CBASB derivatives

**Fig. 10:**
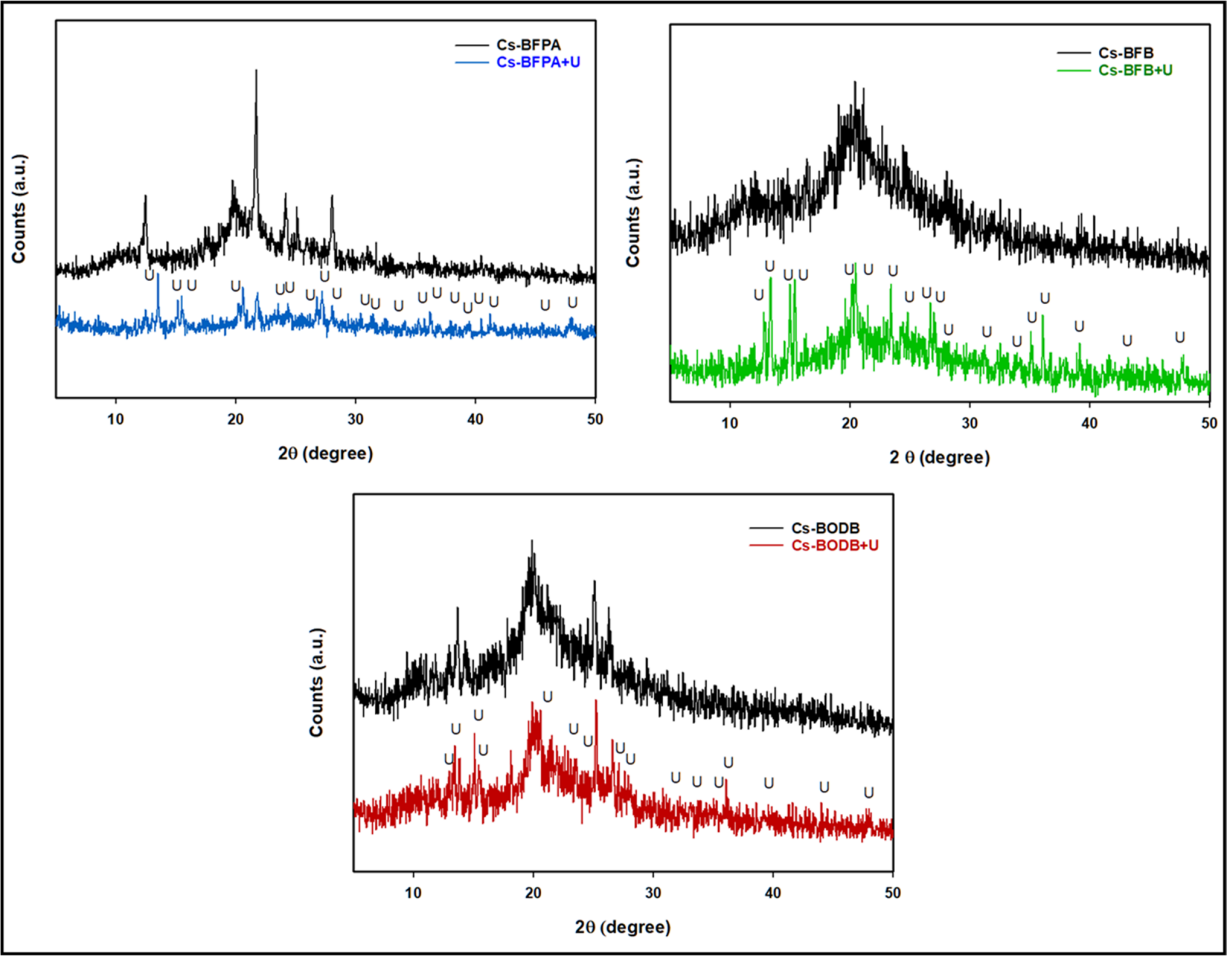
XRD spectra of U complexation with CBASB derivatives

To evaluate the chemical composition of the CBASB derivatives after adsorption, SEM micrographs, EDX, and XRD (Figs. 9, 10) were taken of the material. The results show that the spectra of the three adsorbents contain spots and distinct peaks of U, indicating that U has been adsorbed into the three Cs-*bis*-aldehyde Schiff bases derivatives.

#### Effect of solid/liquid ratio

**Fig. 11:**
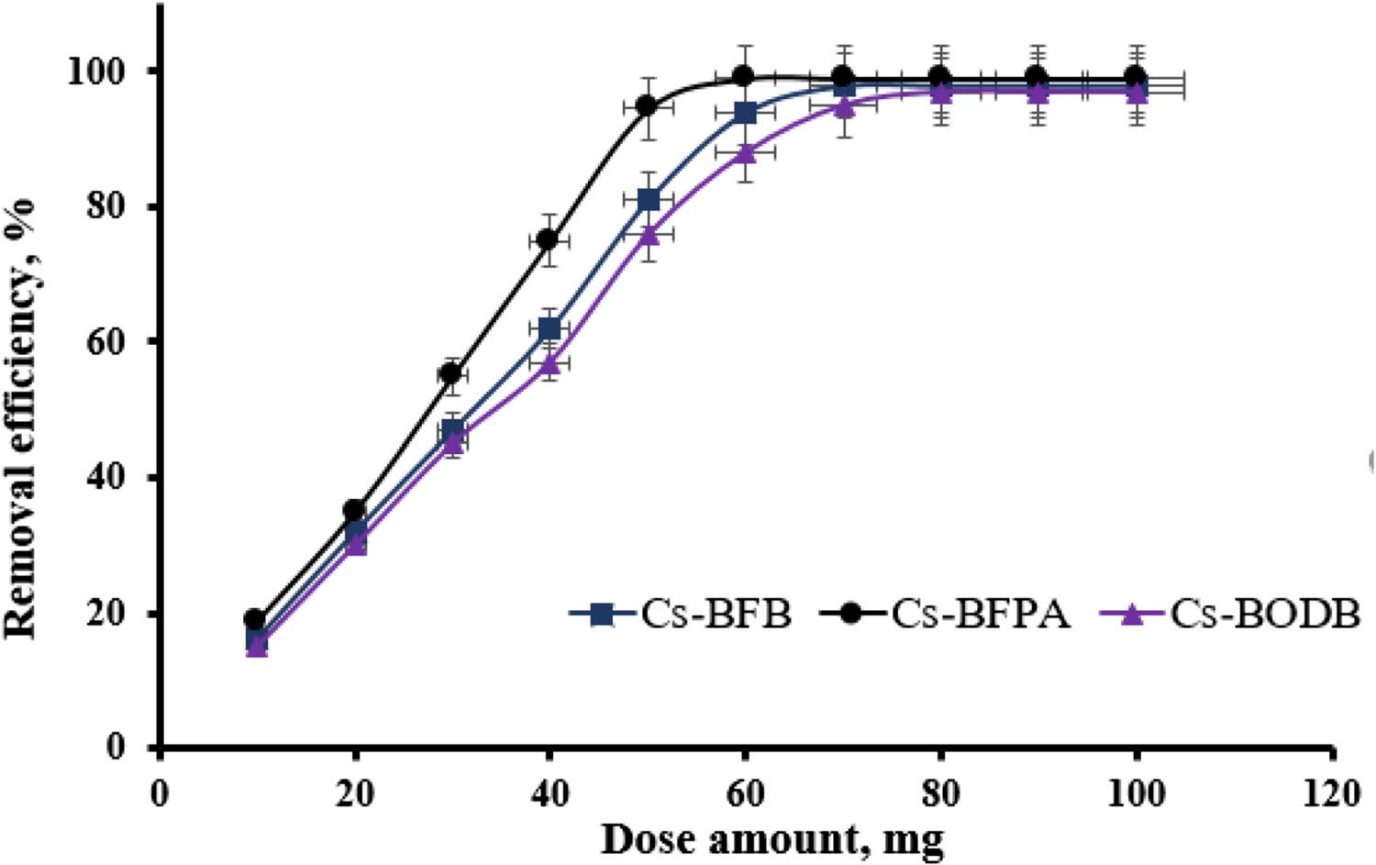
Effect of adsorbent dose on U(VI) adsorption by CBASB derivatives. (Experiment conditions: *C*_0_[U(VI)] = 100.00 ± 0.10 mg/L; pH = 3.00 ± 0.05; *T* = 298.00 ± 1.00 K; *t* = 60 min.)

The solid/liquid ratio adjustment was implemented in order to find out the optimal amount of CBASB adsorbents (Cs-BFPA, Cs-BFB, and Cs-BODB), and the rest of the experimental parameters were kept constant (i.e., contact time was 60 min, adsorption medium volume was 25 mL, and pH value to 3, 100 mg/L metal ion concentration). It can be inferred from Fig. 11 that the adsorption percentage (Ads%) was directly proportional to the amount of the three adsorbents and leveled off at a solid/liquid ratio of 0.06 g which can be attributed to the limited amount of adsorption sites in the initial stages providing a proportional increase of Ads% with the dose. Afterwards, the entrapped U(VI) in the adsorbent will reach a saturation level (dynamic equilibrium) leading to a constant Ads% with increasing the dose.

The results also show that, at equilibrium, Ads% is higher than 94%, 81.0%, and 75% for Cs-BFPA, Cs-BFB, and Cs-BODB, respectively, which indicates that the three adsorbents have excellent adsorption performance for U(VI) removal. Following consideration of the adsorption percentage and the adsorption capacity, an amount of 0.05 g of the three adsorbents was determined to be the optimum amount.

#### Adsorption kinetics and mechanism

The adsorption of metal ions on the material surface can generally be classified into three processes: physical adsorption, ion exchange, and complex adsorption. Physical adsorption occurs through the weak van der Waals interaction and hence is not stable. Adsorption via ion exchange is mainly through the exchange of metal ions with the active protons in the adsorbent. Complex adsorption has been often reported when the adsorbents were polymers containing functional groups such as –NH_2_, –CN, –CO, –OH, –SH, and –SO_3_H. Such groups have a strong chelating ability with metal cations. To investigate the mechanism of adsorption, the adsorption rate was studied, and kinetics models were used to describe the adsorption process.

**Fig. 12:**
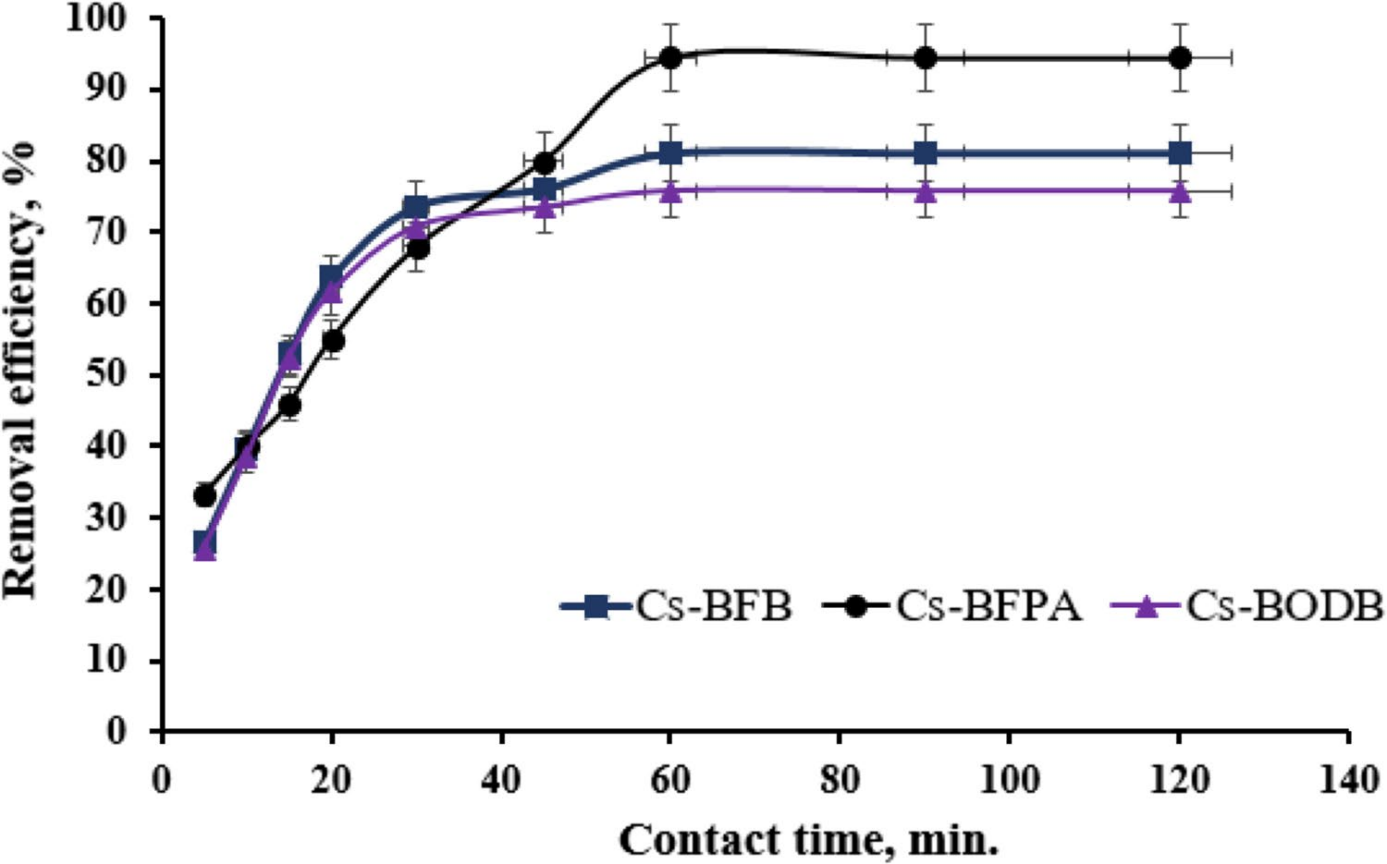
Effect of contact time on U(VI) adsorption by CBASB derivatives (m:V = 0.05 g/25 mL; *C*_0_[U(VI)] = 100.00 ± 0.10 mg/L; pH = 3.00 ± 0.05; *T* = 298.00 ± 1.00 K)

Adsorbent’s efficiency can be predicted by the metal ion separation rate from an aqueous solution which can be inferred from the adsorption kinetics (Yu et al. 2017). The adsorption rate of U(VI) on CBASB derivatives and adsorption capacity are shown in Fig. 12. It can be observed that the adsorption process can be divided into three stages: firstly, the three adsorbents (Cs-BFPA, Cs-BFB, and Cs-BODB) have a lot of vacant binding sites on their surfaces in the first stage, which causes the adsorption capacity and rate to grow quickly. The second stage shows a rise in adsorption capacity and adsorption rate, but a decrease in the gap between adsorption and desorption rates, with maximum values of 94.5%, 81.0%, and 75.8%) for Cs-BFPA, Cs-BFB, and Cs-BODB, respectively, being reached after 60 min. The adsorption process keeps dynamic equilibrium in the third stage, and neither the adsorption rate nor the adsorption capacity will considerably change.

To elucidate the mechanism of U(VI) ion adsorption by CBASB chelators, a pseudo-first and second-order model was used (Eqs. 4 and 5), respectively (Lagergren 1898; Ho and McKay 1999; Orabi et al. 2020, 2021).

4 $$\mathrm{log}\left({q}_{e1}-{q}_{t}\right)=\mathrm{log}{q}_{e1}- \frac{{K}_{1} t}{2.303}$$

5 $$\frac{t}{{q}_{t}} = \frac{1}{{K}_{2 {qe2}^{2}}}+ \frac{t}{{q}_{e2}}$$

where *q*_*e*_ and *q*_*t*_ are the adsorption U amount on the three adsorbents Cs-BFPA, Cs-BFB, and Cs-BODB at equilibrium and *t* (mg/g), respectively; *k*_*1*_ and *k*_*2*_ were the rate constants.

**Fig. 13:**
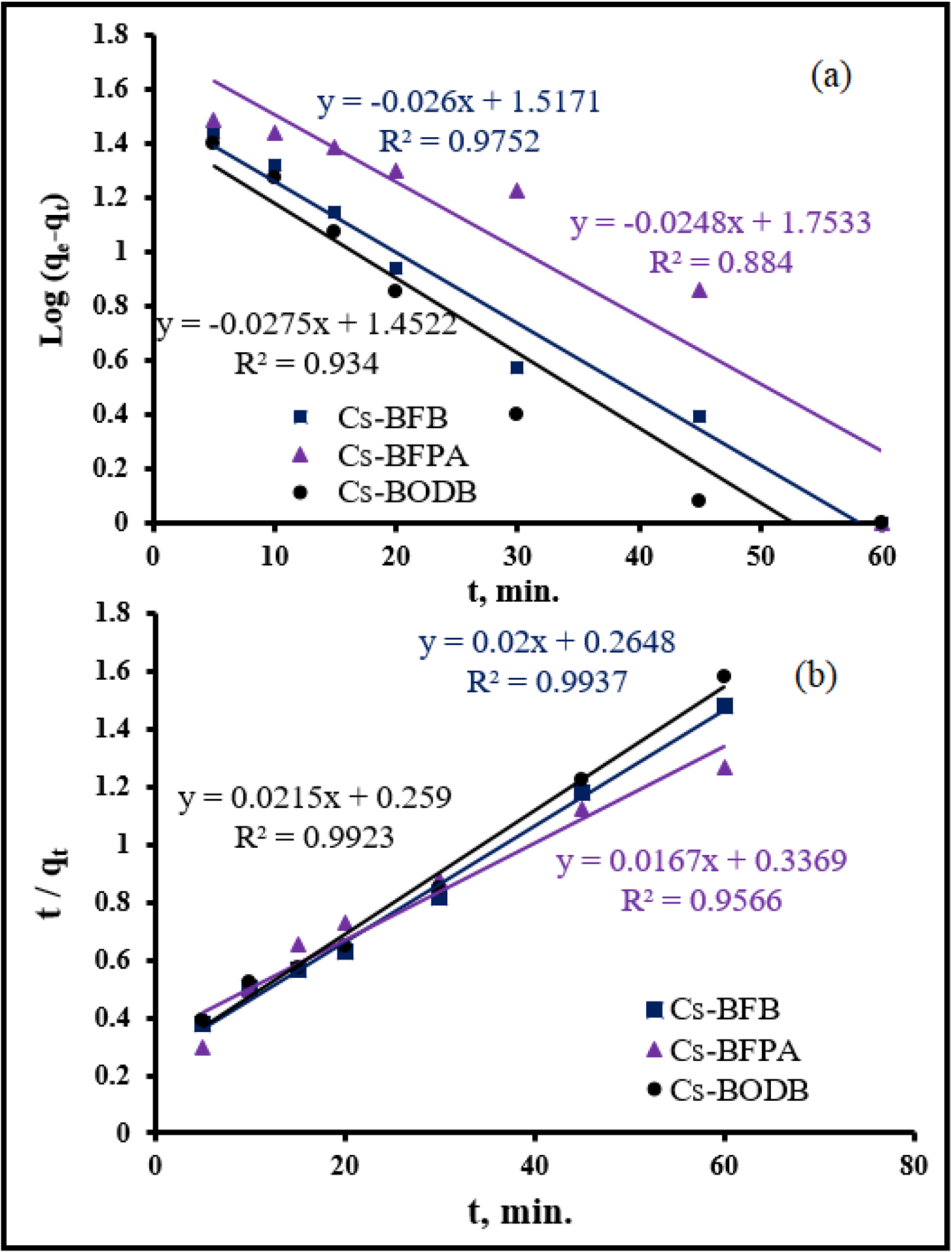
Pseudo-first-order model (**a**) and pseudo-second-order model (**b**) for U(VI) ion adsorption by CBASB derivatives

**Fig. 14:**
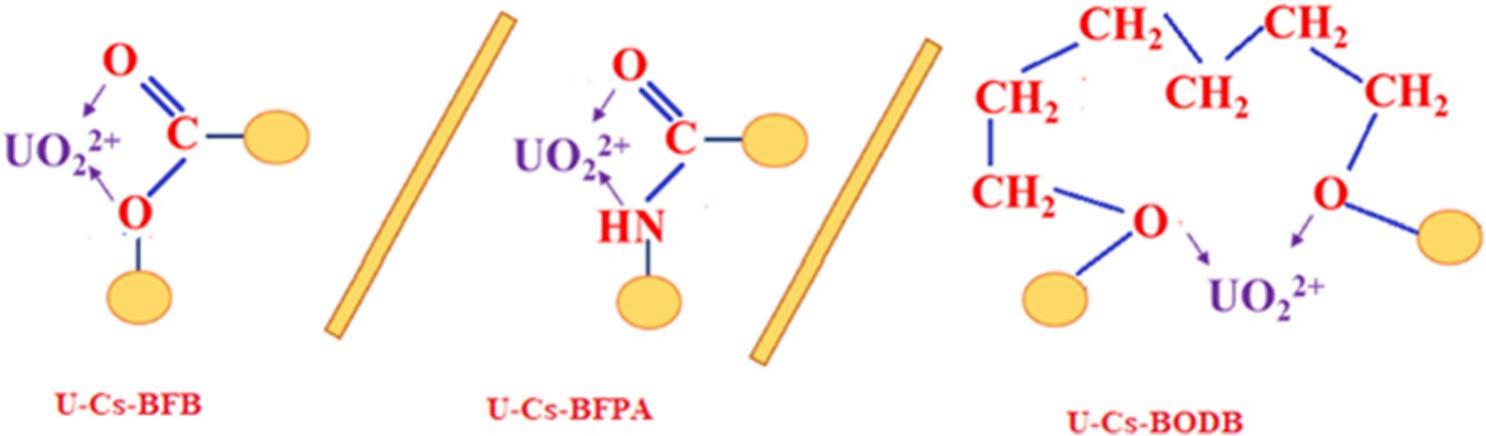
Suggests binding mechanism of functionalized sorbents towards U(VI) ions

The results of the kinetic parameters are shown in Table 3 and Fig. 13a, b. The results show that the dynamic model of pseudo-second-order correlation coefficient (is closer to 1) and *q*_*e*_ is better than the pseudo-first-order kinetics model correlation coefficient, thus, implying that the efficient capture of U(VI) by CBASB derivatives mainly is attributable to surface complexation and the chemisorption of strong forces, rather than ion exchange (Ho and Mckay 2000; Song et al. 2011). It is the availability of CBASB active sites, rather than metal ion concentration, that dictates their adsorption rate (Salameh et al. 2017; Liu et al. 2018). Furthermore, the adsorption rate constants and capacity of U(VI) on these CBASB derivatives are significantly improved compared with their values in the pseudo-first-order kinetics model. In addition, the rate constant of U adsorption using Cs-BFPA is much higher than those with other CBASB derivatives (Cs-BFB and Cs-BODB). This may be due to the presence of amine groups in the Cs-BFPA structure, which have high extractive power, stability, and effective reagent for the separation of uranium from diversified media (Coleman et al. 1958; Crane et al. 2010; Zhu and Cheng 2011; Orabi et al. 2015). The results demonstrated that the nitrogen and oxygen species of CBASB resins had involved in uranium sorption via chemical interaction, which was consistent with the IR variations after the adsorption (Fig. 8). The hard metal ions UO_2_^2+^ might show affinity to hard bases with oxygen and nitrogen donor atoms in the adsorption process according to Pearson (Pearson 1968). Thus, it was inferred that there might be valency forces through sharing of electrons involved in the adsorption behavior between UO_2_^2+^ and the CBASB derivatives. Hence, the chelation mechanism in which –CO and − CN act as a chelating group is proposed for our process as shown in the following Fig. 14.

**Table 3:** Kinetic parameters for the adsorption of U(VI) ions onto synthesized CBASB derivatives

**Adsorbent**	Pseudo-First-order			Pseudo-Second-order		
	*q*_e1_ (mg/g)	*K*_1_ (min^-1^)	*R*^2^	*q*_e2_ (mg/g)	*K*_2_ (g mg^-1^ min^-1^)	*R*^2^
Cs-BFPA	56.66	0.0571	0.884	58.8	0.86×10^-3^	0.957
Cs-BFB	32.89	0.0598	0.975	50.0	1.51×10^-3^	0.994
Cs-BODB	28.33	0.0633	0.934	46.5	1.78×10^-3^	0.992

#### Effect of the initial concentration of U(VI)

**Fig. 15:**
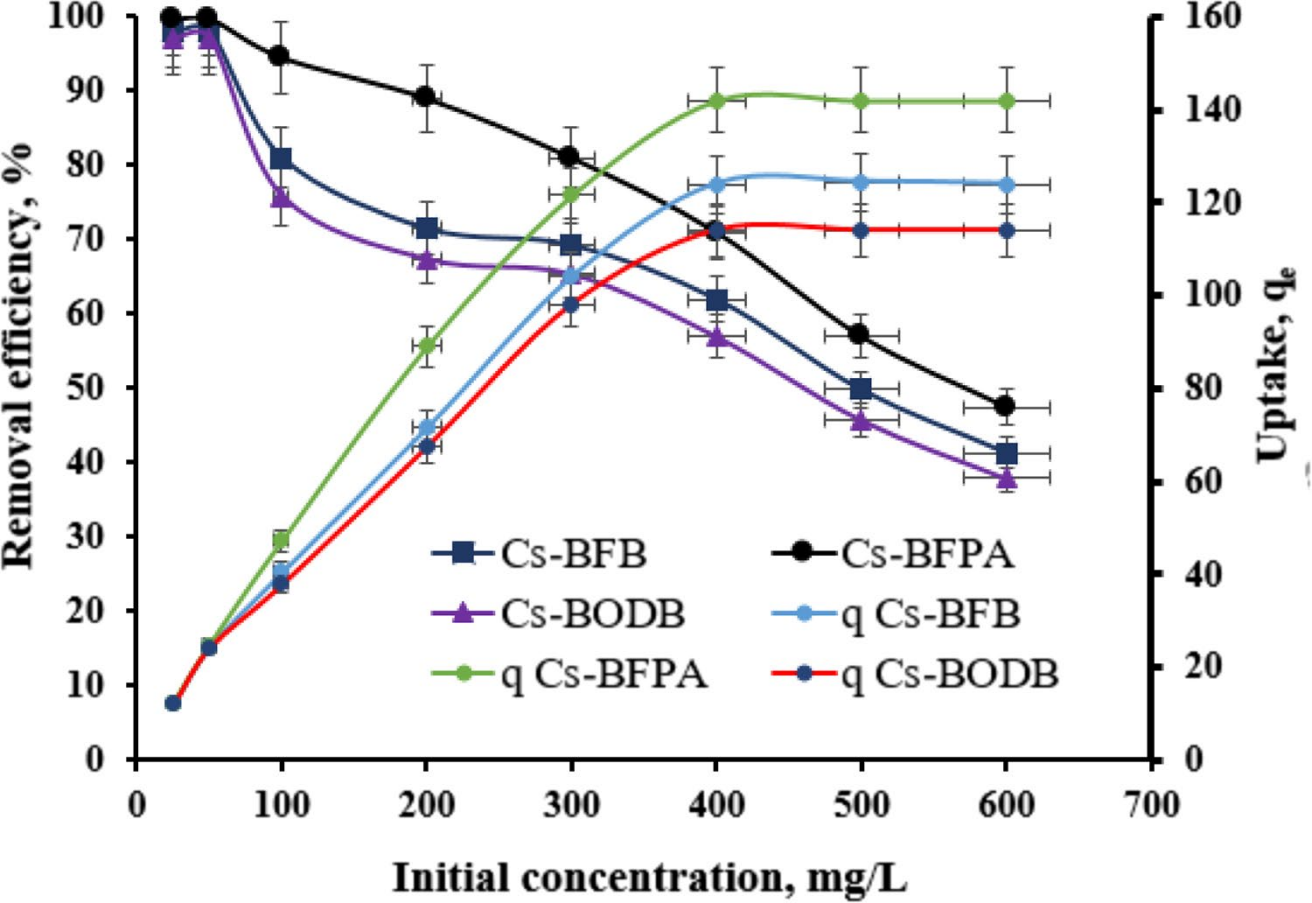
Effect of the initial concentration of U(VI) to the adsorption process. (Experiment conditions: temperature = 25.00 °C; *C*_0_[U(VI)] = 25.00 ~ 600.00 mg/L; solid/liquid ratio = 0.05 g/25 mL; pH = 3.00 ± 0.05; *t* = 60 min.)

Figure 15 shows the relationship between the initial concentration of U(VI) and the relative adsorption capacity of CBASB derivatives (Cs-BFPA, Cs-BFB, and Cs-BODB) at room temperature. At the initial stage, the adsorption amount reached a maximum value and decreased with increasing the concentration of U(VI), as shown in Fig. 15. The results in Fig. 15 exposed that as the initial concentrations increased, it led to increasing the uranium uptake that reached a maximum loading at 400 mg/L uranium concentration. After that, the overloaded uranium kept constant. It represents that the studied adsorbents reached the maximum packing capacity (saturation capacity), because the mobility of UO_2_^2+^ in the solutions is the highest and the whole active sites of the adsorbents are saturated and blocked with uranyl ions. The maximum adsorption capacity of the three adsorbents for U(VI) reaches 142, 124, and 114 mg/g for Cs-BFPA, Cs-BFB, and Cs-BODB, respectively. Having underlined the above-mentioned adsorptive features, the sorption potential of Cs-BFPA, Cs-BFB, and Cs-BODB was compared with those acquired from the literature in Table 4. A comparison of *q*_*max*_ values demonstrates that synthesized Cs-BFPA, Cs-BFB, and Cs-BODB exhibit a decent aptitude for uranium adsorption. The sorption capacity of CBASB derivatives (Cs-BFPA, Cs-BFB, and Cs-BODB) for uranium(VI) was significantly higher than those acquired from the literature in Table 3 (Oren et al. 2000; Venkatesan et al. 2004; Wang et al. 2009; Kadous et al. 2010; Morsy 2015; Orabi et al. 2016, 2020; Ali and Nouh 2019; Fouad et al. 2019; Xiao-teng et al. 2019; Orabi et al. 2021). Also, as shown in Table 4, although the sorption capacity of CBASB derivatives resin is lower than some novel adsorbents, e.g., graphene oxide (Sun et al. 2015), their regeneration has not been considered. In real applications, the reusability of adsorbents is important to reduce the running cost and to decrease the environmental impacts. Compared with carbon sphere@layered double hydroxide (desorption time 12 h) (Wang et al. 2018), montmorillonite@carbon composite (desorption time 8 h) (Zhang et al. 2015), carbonaceous nanofibers (desorption time 1 h) (Sun et al. 2016), Fe3O4@MnOx (desorption time 6 h) (Song et al. 2019), and Polyethyleneimine-alkali-biochar (desorption time 12 h) (Wang et al. 2020), which still showed high removal efficiency towards uranium(VI) even after several cycles of regeneration experiments, it took much less time to regenerate for CBASB derivatives.

**Table 4:** Comparison of the uranium sorption capacity of synthesized Cs-BFPA, Cs-BFB, and Cs-BODB with other sorbents

Sorbent	Uranium sorption capacity (mg /g)	References
Silica modified with rhodamine-B	35.00	(Ali and Nouh 2019)
alizarin red S -impregnated XAD-2010	20.20	(Fouad et al. 2019)
polyelectrolyte N-vinyl-2-pyrrolidone-g-tartaric acid	53.20	(Oren et al. 2000)
Chitosan@attapulgite composite	22.48	(Morsy 2015)
Cellulose impregnated with amine	54.50	(Orabi et al. 2016)
Grafted polystyrene resin	41.76	(Kadous et al. 2010)
Acrylic fiber waste/sargassum	62.00	(Orabi et al. 2020)
Modified chitosan	49.00	(Wang et al. 2009)
Modified rice Stem	18.00	(Xiao-teng et al. 2019)
Silica gel-amide	28.98	(Venkatesan et al. 2004)
Polysulfone/chitosan grafted p-phenylenediamine	44.00	(Orabi et al. 2021)
Cellulose acetate/chitosan grafted p-phenylenediamine	39.00	(Orabi et al. 2021)
Carbon sphere@layered double hydroxide	156.60	(Wang et al. 2018)
Polyethyleneimine-alkali-biochar	212.70	(Wang et al. 2020)
Montmorillonite@carbon composite	66.20	(Zhang et al. 2015)
Fe_3_O_4_@MnO_x_	244.60	(Song et al. 2019)
Carbonaceous nanofibers	125.00	(Sun et al. 2016)
Cs-BFPA	142.00	This work
Cs-BFB	124.00	This work
Cs-BODB	114.00	This work

**Fig. 16:**
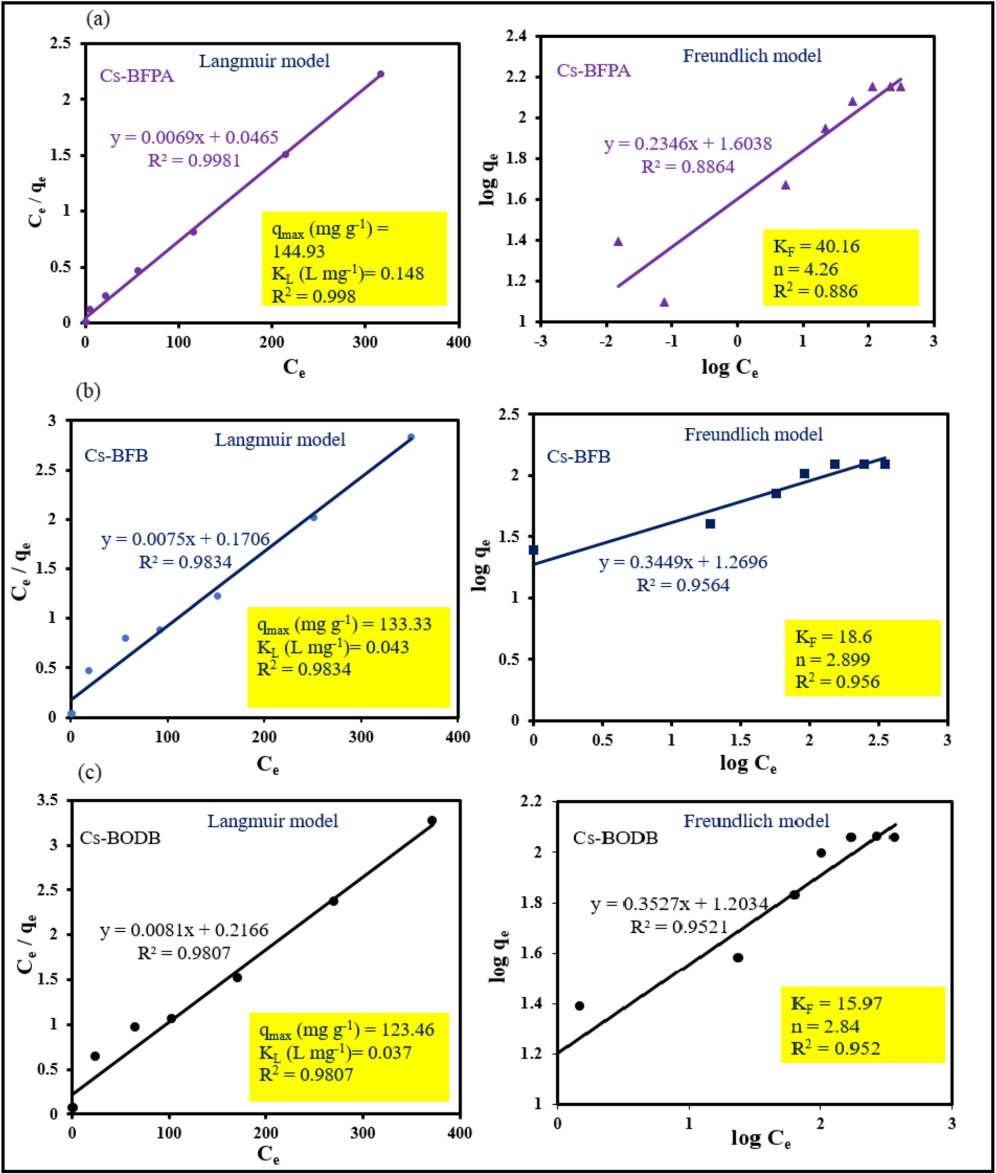
Langmuir and Freundlich models of U adsorption by Cs-BFPA (**a**), Cs-BFB (**b**), and Cs-BODB (**c**)

When considering adsorption isotherms, both homogeneous monolayer and heterogeneous adsorbent surfaces can be used in Langmuir and Freundlich models, which are described by Eqs. (6) and (7), respectively (Srinivasan et al. 1997; Foo and Hameed 2010; Orabi et al. 2021):

6 $$\frac{{C}_{e}}{{q}_{e}} = \frac{Ce}{{q}_{max}}+ \frac{1}{{K}_{L {q}_{max}}}$$

7 $$\mathrm{log}{q}_{e}=\mathrm{log}{K}_{f}+ \frac{{logC}_{e} }{n}$$

where *q*_max_ is the maximum adsorption amount at equilibrium in Langmuir isotherm (mg/g) and *K*_L_ is the adsorption equilibrium constant (L/mg). *K*_f_ is the adsorption amount (mg/g), and *n* is the Freundlich constant related to surface heterogeneity. Figure 16a, b, and c show that the Langmuir model fits better than the Freundlich model, suggesting that the adsorption of U(VI) cation proceeds via the monolayer mechanism (Langmuir model). Based on the Langmuir models, the theoretical maximum uranium adsorption of Cs-BFPA, Cs-BFB, and Cs-BODB was found to be 144.9, 133.3, and 123.5 mg/g, respectively. Freundlich isotherm model is quite different from the experimental adsorption capacity. Correlation fitting results are collected in Fig. 16a, b, and c.

#### Effect of temperature (thermodynamics studies)

**Fig. 17:**
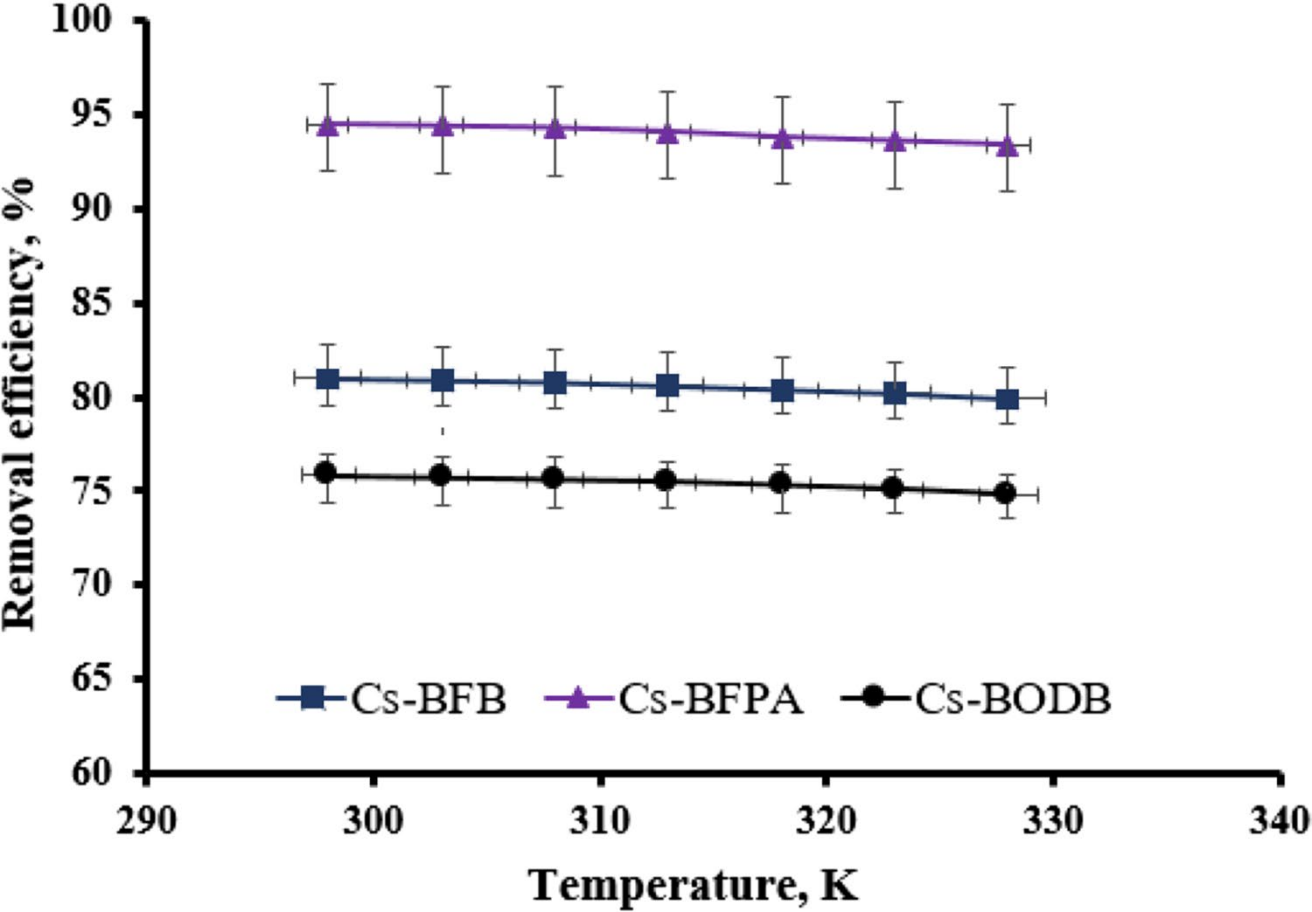
Effect of temperature on the adsorption efficiency of U(VI) using synthesized CBASB derivatives adsorbents

With synthesized CBASB derivatives (Cs-BFPA, Cs-BFB, and Cs-BODB) as test samples, the adsorption procedure was run in batches at various temperatures between 298 and 328 K in order to determine the impact of temperature on U(VI) adsorption from a solution of pH = 3. The other parameters were as follows: *C*_0_[U(VI)] = 100.00 mg/L; solid/liquid ratio = 0.05 g/25 mL; *t* = 60 min. Figure 17 illustrates that, at the scale under consideration, heating has only a minor effect on adsorption. Thus, 25 °C (298 K) might be considered the ideal temperature for U adsorption tests.

**Fig. 18:**
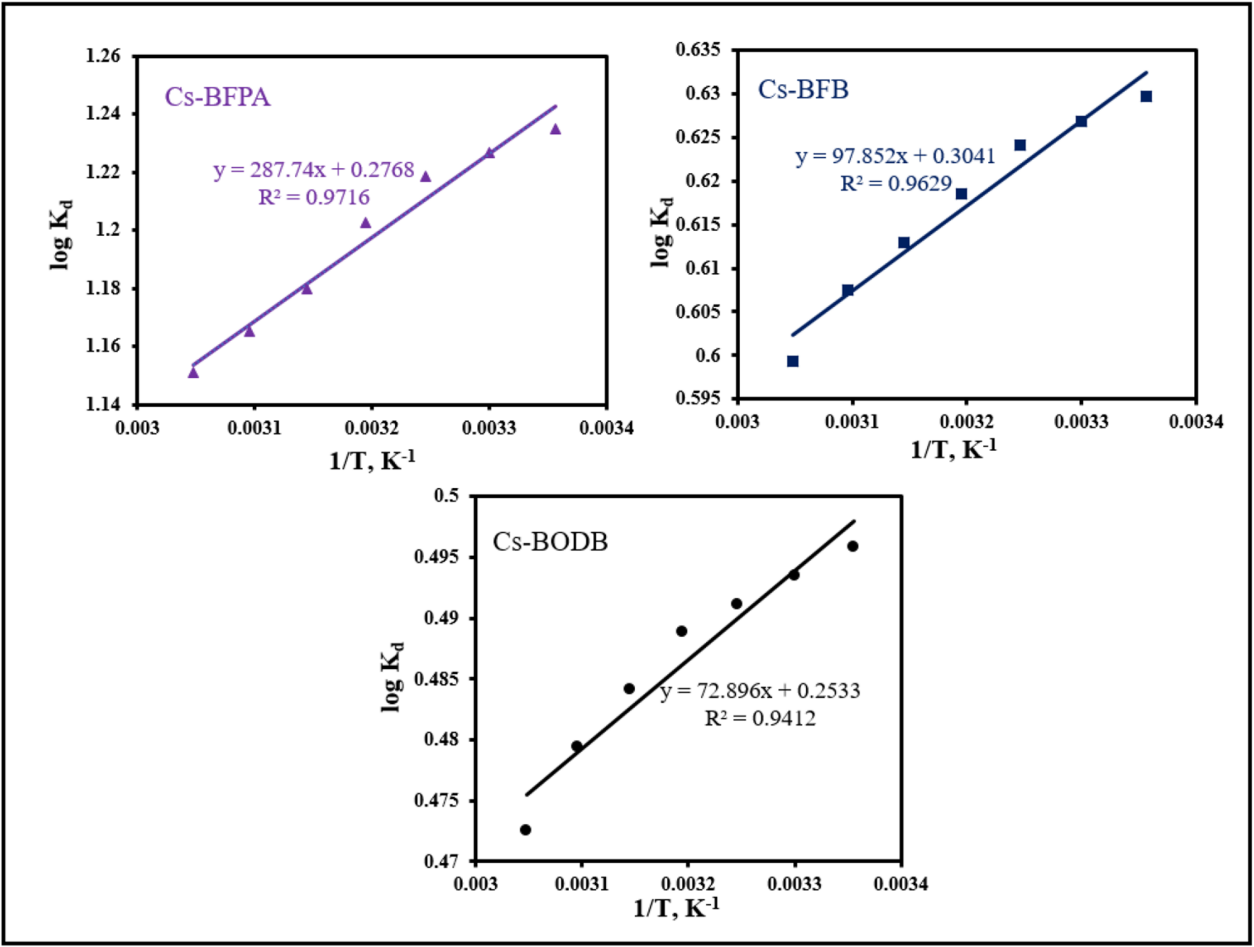
The plot of log *K*_*d*_ versus *1/T* of the U(VI) adsorption upon CBASB derivatives (Cs-BFPA, Cs-BFB, and Cs-BODB)

Calculating the change in relevant thermodynamic parameters can be done with the help of the equation developed by Van’t Hoff.

8 $$\mathrm{log}{K}_{d}=\Delta S/2.303R- \Delta H/2.303RT$$

9 $$\Delta G=\Delta H- T\Delta S$$

where *R* is the ideal gas constant. The values of *K*_*d*_ at the different temperatures (Fig. 18) were used to calculate the thermodynamic parameters (Table 5) for the adsorption of U(VI) ions on CBASB derivatives (Cs-BFPA, Cs-BFB, and Cs-BODB).

**Table 5:** Thermodynamic parameters for the adsorption of U(VI) ions onto CBASB derivatives

Adsorbent	*ΔG* (kJ·mol^−1^)	*ΔH* (kJ·mol^−1^)	*ΔS* (J·mol^−1^·K^−1^)
Cs-BFPA Cs-BFB Cs-BODB	− 7.08 − 3.60 − 2.84	− 5.50 − 1.87 − 1.39	5.30 5.82 4.85

The presence of negative values for *ΔG* (− 7.08, − 3.6, and − 2.84 kJ·mol^−1^ for Cs-BFPA, Cs-BFB, and Cs-BODB, respectively) implies that the binding process of CBASB derivatives with U(VI) is spontaneous Because each of the three adsorbents has a value of *ΔS* that is more than zero, it can be deduced that the degree of randomness in the U(VI) bonding with CBASB derivatives rises. *ΔH* values of the three adsorbents are less than zero, which also proves that the adsorption process for all adsorbents is an exothermic reaction (Srinivasan et al. 1997; Khawassek et al. 2018; Ahmad 2020; Orabi et al. 2021).

### Effect of co-existing ions

There are many kinds of metal ions in the waste liquid system to be treated, so it is important to study the specific adsorption of target metal ions by adsorption materials (Cs-BFPA, Cs-BFB, and Cs-BODB). In this experiment, common metal ions (Fe^3+^, Al^3+^, Mn^2+^, Cu^2+^, Co^2+^, Mg^2+^, Zn^2+^, Pb^2+^, Ni^2+^, Cd^2+^) in waste liquid were selected as co-existing competitive ions, and the concentration of all the metal ions was 10 mg in a solution of 100 mL. The changes in the concentration of each metal ion were determined by ICP-OES. The results in Table 6 indicate a descending order of metal ions of U > Al > Fe > Mg > Mn > Zn > Cu > Co > Ni > Pb and Cd loaded by the three synthesized adsorbents. As shown in Table 6, under the set conditions, the adsorption percentage of U(VI) by Cs-BFPA, Cs-BFB, and Cs-BODB can reach 94.5%, 81.0%, and 75.8%, respectively, while the Ads% of other metal ions is less than 31%. In addition, the adsorption data showed that the three novel cross-linked chitosan *bis*-aldehyde derivatives possessed good selectivity of U(VI) in the order of Cs-BFPA > Cs-BODB > Cs-BFB. The separation factors of U to impurities using Cs-BFPA are much higher than those with other CBASB derivatives (Cs-BFB and Cs-BODB). This may be due to the presence of amine groups in Cs-BFPA, which have high extractive power, stability, selectivity, and effective reagent for the separation of uranium from diversified media (Coleman et al. 1958; Crane et al. 2010; Zhu and Cheng 2011; Orabi et al. 2015).

**Table 6:** Effect of some interfering metal ions upon Cs-BFPA, Cs-BFB, and Cs-BODB adsorbents

Metal ions	Loaded Conc, mg/g			Separation factor U/M		
	Cs-BFPA	Cs-BFB	Cs-BODB	Cs-BFPA	Cs-BFB	Cs-BODB
U	94.5	81.0	75.8	17.2	4.3	3.1
Al	27.0	30.6	29.0	46.4	9.7	7. 5
Fe	21.2	27.0	24.2	63.6	11.5	9.8
Mg	10.3	17.2	14.3	149.4	20.6	18.7
Zn	5.8	7.6	6.2	281.6	51.9	47.4
Cu	5.0	6.3	5.8	324.2	63.6	51.3
Co	4.8	5.3	5.0	343.6	76.1	59.5
Mn	4.5	5.2	5.0	365.5	77.5	59.5
Ni	4.4	4.9	4.7	373.5	82.7	63.5
Pb	4.1	4.9	4.4	402.3	82.7	68.0
Cd	4.0	4.6	4.0	412.9	88.4	75.5

### Elution and reusability

Quantitative desorption of U(VI) was performed using a variety of eluting agents (1 M) from the loaded sorbents under investigation in this study (Cs-BFPA, Cs-BFB, and Cs-BODB). Nitric acid (HNO_3_), hydrochloric acid (HCl), and sulfuric acid (H_2_SO_4_) have been used as desorbents for this purpose. Compared with the three acids, sulfuric acid has the best desorption effect on U(VI) ions (98.7% for Cs-BFPA, 98.5% for Cs-BFB, and 98% for Cs-BODB) followed by HNO_3_ (95.8% for Cs-BFPA, 95.3% for Cs-BFB, and 95% for Cs-BODB), and the minimal values by HCl (85.2% for Cs-BFPA, 80.3% for Cs-BFB, and 78% for Cs-BODB).

**Fig. 19:**
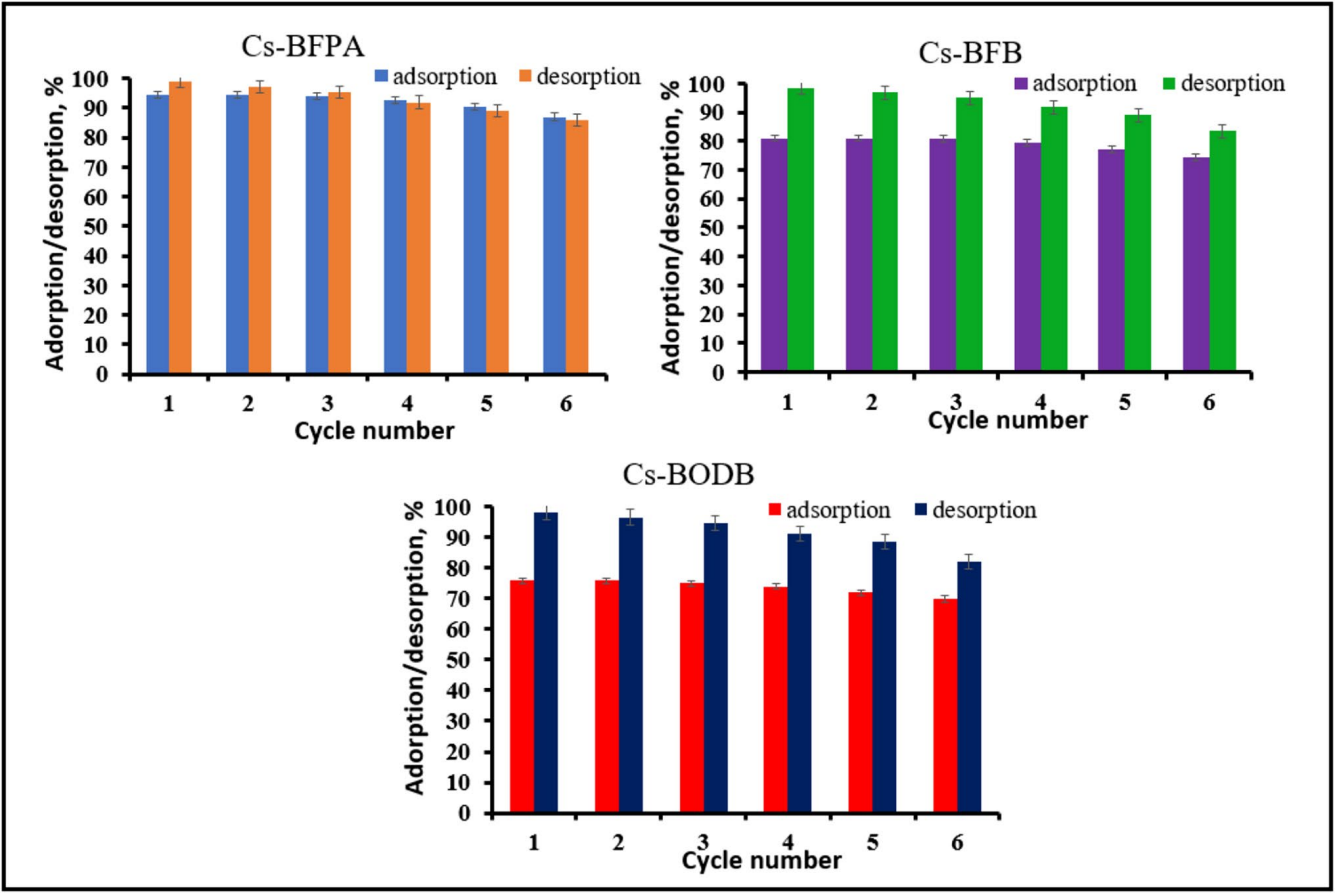
The adsorbed and desorbed of U(VI) ions efficiency as adsorption–desorption cycle function

According to the results of a prior study, the regeneration and reusability of CBASB adsorbents were essential characteristics for determining whether or not they could be used in practical industrial applications. During the regeneration process, the U(VI) ions packed into the three adsorbents were immersed in a regeneration solution containing 1 M/L H_2_SO_4_. It can be shown in Fig. 19 that the adsorption–desorption efficiencies of U(VI) on CBASB derivatives declined with the number of cycles performed. After six cycles of CBASB derivatives reusability, desorption efficiencies recovered to up to 85.8% for Cs-BFPA, 83.3% for Cs-BFB, and 82% for Cs-BODB of their initial values, indicating a significant improvement. As a result, it was determined that CBASB derivatives, because of their long-term consistency, were accepted as a brilliant reusable adsorbent for the very efficient removal of U(VI).

### Case study

It was decided to conduct a case study of sorption utilizing 1L of two waste solutions (a and b) with U concentrations of 76 mg/ L and 54 mg/ L, respectively, in contact with 5 g of synthesized CBASB derivatives (Cs-BFPA, Cs-BFB, and Cs-BODB) at temperatures of 298 K, time constant of 1 h, and pH values of 3. Following the completion of the balancing, the solution was filtered, and the concentrations of U were determined. A total of 91 and 89% of the removal efficiency of Cs-BFPA were discovered, respectively. In contrast, the removal efficiency was 76% and 74.5%, respectively, for Cs-BFB. But, in the case of Cs-BODB, the removal efficiency was 72% and 71.7%, respectively. Reduced efficiency in removing U by synthesized Cs-BFPA, Cs-BFB, and Cs-BODB following contact with the waste solution may be attributed to the rivalry in the nuclear waste sample between the various elements (particularly iron) and U ions. A solution of 1 M H_2_SO_4_ was used to desorb the sorbed U. As a consequence of the research, it was discovered that the synthetic CBASB derivatives were capable of successfully removing and separating U from the actual waste solution.

## Conclusions

Covalently cross-linked chitosan Schiff bases were synthesized using by making use of aromatic bis-aldehyde linkers that had variable spacer functions, and the structures of the produced Schiff bases were characterized by ^1^H NMR, FTIR, XRD, SEM, and TGA. The adsorption of U via synthesized three adsorbents (Cs-BFPA, Cs-BFB, and Cs-BODB) was examined in this study. Pseudo-second-order kinetic model will explain the adsorption kinetics of U on the three synthesized adsorbents. The Langmuir model analysis results show that the theoretical maximum adsorption capacities of U cations are up to 144.9 mg/g for Cs-BFPA, 133.3 mg/g for Cs-BFB, and 123.5 mg/g for Cs-BODB. The thermodynamic analyses confirm the exothermic nature and the spontaneity of the adsorption process. The kinetic study of the adsorption suggests that the rate-limiting step may be chemical adsorption. IR and SEM spectra confirm the chelation of U cations with the three synthesized adsorbents. The adsorbent materials (Cs-BFPA, Cs-BFB, and Cs-BODB) can be selected in the long term and accepted as brilliant reusable adsorbents for highly efficient removal of U under the premise of ensuring the adsorption capacity. All these results clearly indicate that the three synthesized adsorbents are alternative and efficient adsorbents for the recovery of precious U from waste solutions.

## Data Availability

All relevant data and material are presented in the main paper.
